# The effect of an intraoperative patient-specific, surgery-specific haemodynamic algorithm in improving textbook outcomes for hepatobiliary–pancreatic surgery: a multicentre retrospective study

**DOI:** 10.3389/fsurg.2024.1353143

**Published:** 2024-05-27

**Authors:** Bradly Carp, Laurence Weinberg, Luke R. Fletcher, Jake V. Hinton, Adam Cohen, Hugh Slifirski, Peter Le, Stephen Woodford, Shervin Tosif, David Liu, Vijaragavan Muralidharan, Marcos V. Perini, Mehrdad Nikfarjam, Dong-Kyu Lee

**Affiliations:** ^1^ Department of Anaesthesia, Austin Health, University of Melbourne, Melbourne, VIC, Australia; ^2^ Department of Critical Care, University of Melbourne, Parkville, VIC, Australia; ^3^ Department of Surgery, Austin Health, University of Melbourne, Melbourne, VIC, Australia; ^4^ Data Analytical Research Unit, Austin Health, Melbourne, VIC, Australia; ^5^ Department of Anesthesiology and Pain Medicine, Dongguk University Ilsan Hospital, Goyang, Republic of Korea

**Keywords:** textbook outcome, complications, haemodynamic, algorithm, hepatobiliary–pancreatic, surgery

## Abstract

**Background:**

The concept of a “textbook outcome” is emerging as a metric for ideal surgical outcomes. We aimed to evaluate the impact of an advanced haemodynamic monitoring (AHDM) algorithm on achieving a textbook outcome in patients undergoing hepatobiliary–pancreatic surgery.

**Methods:**

This retrospective, multicentre observational study was conducted across private and public teaching sectors in Victoria, Australia. We studied patients managed by a patient-specific, surgery-specific haemodynamic algorithm or via usual care. The primary outcome was the effect of using a patient-specific, surgery-specific AHDM algorithm for achieving a textbook outcome, with adjustment using propensity score matching. The textbook outcome criteria were defined according to the International Expert Delphi Consensus on Defining Textbook Outcome in Liver Surgery and Nationwide Analysis of a Novel Quality Measure in Pancreatic Surgery.

**Results:**

Of the 780 weighted cases, 477 (61.2%, 95% CI: 57.7%–64.6%) achieved the textbook outcome. Patients in the AHDM group had a higher rate of textbook outcomes [*n* = 259 (67.8%)] than those in the Usual care group [*n* = 218 (54.8%); *p* < 0.001, estimated odds ratio (95% CI) 1.74 (1.30–2.33)]. The AHDM group had a lower rate of surgery-specific complications, severe complications, and a shorter hospital length of stay (LOS) [OR 2.34 (95% CI: 1.30–4.21), 1.79 (95% CI: 1.12–2.85), and 1.83 (95% CI: 1.35–2.46), respectively]. There was no significant difference between the groups for hospital readmission and mortality.

**Conclusions:**

AHDM use was associated with improved outcomes, supporting its integration in hepatobiliary–pancreatic surgery. Prospective trials are warranted to further evaluate the impact of this AHDM algorithm on achieving a textbook impact on long-term outcomes.

## 1 Introduction

Despite the advancements in surgical techniques, anaesthesia, and perioperative care for patients undergoing hepatobiliary–pancreatic (HBP) surgery, morbidity remains significant owing to the large fluid shifts, extensive abdominal dissection, intraoperative bleeding, and prolonged operative times (1, 2). Notably, up to 40% of patients undergoing HBP surgery experience severe complications (Clavien–Dindo >III) (3–5).

First described in the field of gastrointestinal cancer surgery, the concept of a “textbook outcome” (TO) is emerging as a robust metric of an ideal surgical outcome for patients undergoing HBP surgery (6, 7). A TO represents the optimal course following a surgical episode, achieved when prespecified parameters are fulfilled according to an all-or-none principle (8). The TO metric also offers a comprehensive assessment of patient-level outcomes and hospital performance, serving as a valuable benchmarking tool for comparing the effectiveness of emerging therapeutic interventions and evaluating variations between healthcare institutions (9–11).

Multiple definitions of TO in HBP surgery have been reported (12); recently, however, the “International Expert Delphi Consensus on Defining Textbook Outcome in Liver Surgery” (13) and “Nationwide Analysis of a Novel Quality Measure in Pancreatic Surgery” (14) reported consensus definitions for liver and pancreas surgery, respectively (see Supplementary Table S1).

The effectiveness of advanced haemodynamic monitoring (AHDM) in patients undergoing HBP surgery remains inconclusive, with multiple studies demonstrating conflicting effects on length of stay (LOS), cost, complications, morbidity, and mortality (15–21). However, no studies to date have investigated the effects of AHDM on the TO of patients undergoing HBP surgery.

We conducted a multicentre, retrospective, propensity score–matched study to evaluate the effects of AHDM on the TO of these patients. Our algorithm is patient-specific, surgery-specific, and seeks to individualise fluid and vasoactive therapies to support circulatory homeostasis and end-organ perfusion. We hypothesised that our AHDM would be associated with a higher rate of TO for patients undergoing HBP surgery.

## 2 Methodology

This retrospective multicentre observational study was conducted at three hospitals in the state of Victoria, Australia. All three hospitals are served by the same HBP surgeons and anaesthesiologists. The Austin Health Human Research Ethics Committee approved this study (approval number 2022/Austin/34) and waived the requirement for participant consent. The study protocol was registered in the Australian New Zealand Clinical Trials Registry (ANZCTR: 324237676) and reported following the Strengthening the Reporting of Observational Studies in Epidemiology guidelines (22).

### 2.1 Inclusion and exclusion criteria

Patients who underwent elective liver or pancreatic resection surgery via a laparoscopic, open, or hybrid approach between January 2011 and December 2022 were screened for inclusion. Major liver surgery was defined as the resection of four or more liver segments. Minor liver surgery was defined as the resection up to and including three segments, excluding liver biopsies. Pancreatic procedures were categorised into either Whipple procedures or other pancreatic procedures that included central, distal, and total pancreatectomies. All patients aged ≥18 years and managed by a patient-specific, surgery-specific intraoperative AHDM algorithm (AHDM group) or usual care (Usual care group) were included. Exclusion criteria included patients undergoing emergency surgery, liver transplantation, any non-HBP concomitant procedure (e.g., combined bowel resection), or patients requiring venovenous bypass. Patients who participated in previous randomised clinical trials where a protocolised AHDM algorithm was applied were also excluded.

### 2.2 Routine care for all patients

All patients underwent a standardised enhanced recovery after surgery (ERAS) protocol implemented across all institutions. Perioperative management of anaemia, glycaemic control, and optimisation of comorbidities was undertaken by dedicated multidisciplinary teams using previously reported ERAS protocols (19). While there have been small refinements to the ERAS pathways over the study period, the fundamental principal and framework underpinning them have been unchanged.

All patients received invasive haemodynamic monitoring that included an arterial line and a central venous catheter to measure mean arterial pressure (MAP) and central venous pressure (CVP), respectively. Use of volatiles or total intravenous anaesthesia, modality of analgesia, use of processed electroencephalography and cerebral oximetry monitoring, and ventilation strategies were at the discretion of the attending anaesthesiologist. Most patients were electively admitted to the intensive care unit (ICU) for routine postoperative monitoring, which did not routinely involve the use of any AHDM devices or algorithms.

### 2.3 Usual care group

In patients receiving usual care, the attending anaesthesiologist had clinical discretion over fluid administration and use of vasoactive medications using the information that was available to them. A FloTrac sensor was not used in any of the patients in this group.

### 2.4 AHDM group

Patients in the AHDM group had a FloTrac sensor (Edwards Lifesciences, Irvine, CA, USA) connected to their arterial line, which provided additional haemodynamic variables including cardiac output, stroke volume (SV), stroke volume variation (SVV), and systemic vascular resistance. These were calculated continuously and presented to the attending anaesthesiologist via the EV1000 or HemoSphere clinical platforms (Edwards Lifesciences, Irvine, CA, USA).

**Figure 1:**
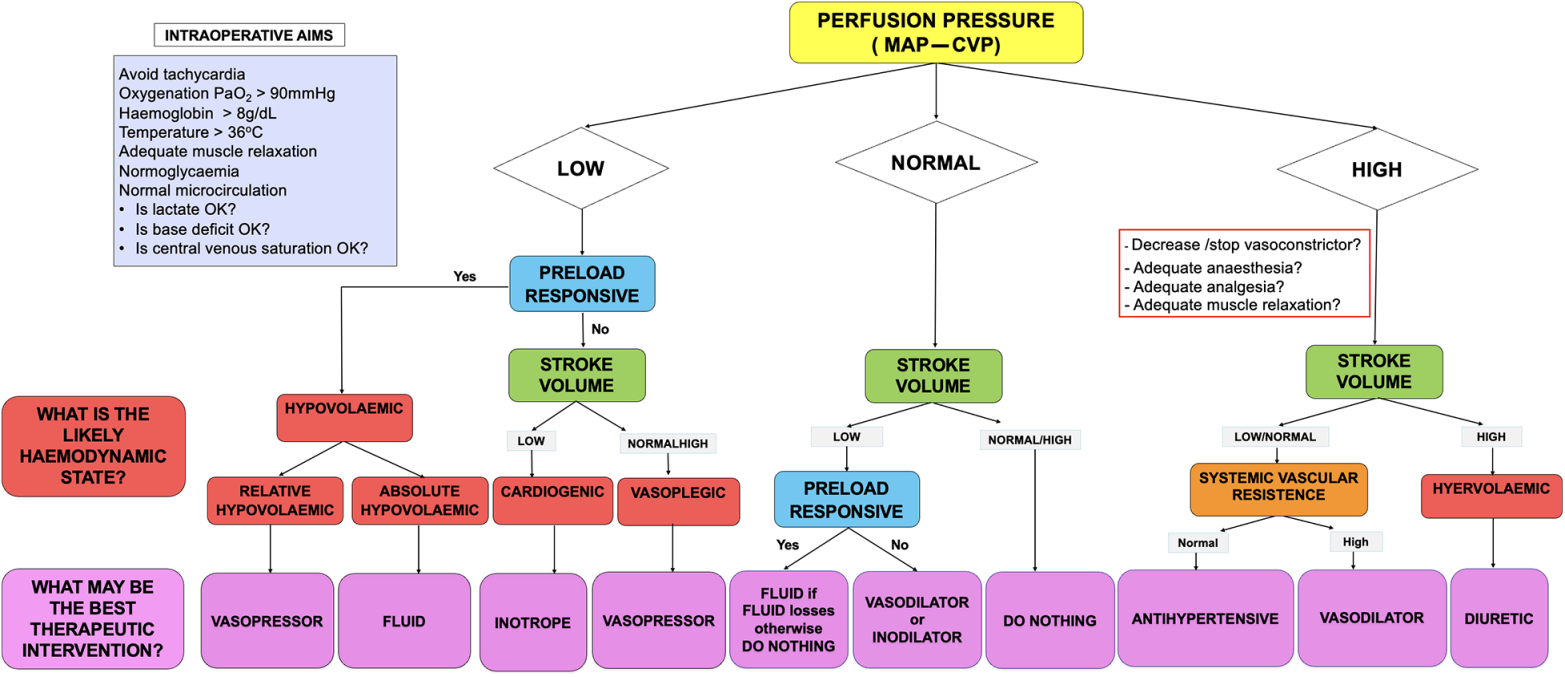
Advanced haemodynamic monitoring algorithm.

Fluid administration and the use of vasoactive therapies were guided by a patient-specific, surgery-specific AHDM algorithm that sought to maintain circulatory homeostasis and end-organ perfusion. The AHDM algorithm prescribes haemodynamic targets for perfusion pressure (MAP − CVP), SV, and SVV, which were defined preoperatively and defended intraoperatively (Figure 1).

Baseline MAP was estimated from preoperative ambulatory non-invasive mean blood pressure measurements. Baseline CVP was measured with the patient in the supine position, immediately post central venous catheter insertion, and prior to surgery commencing. Intraoperative perfusion pressure was defended to within 20% of the baseline and to greater than 65 mmHg. Baseline SV was measured from preoperative echocardiography or estimated as 1.0–1.5 ml/kg. Intraoperative SV was defended to within 20% of the baseline. Preload responsiveness was defined by a patient-specific, surgery-specific SVV target. For patients undergoing liver resection surgery, the intraoperative SVV target was set between 20% and 25% during the dissection and resection phases and adjusted to 20% post resection. For patients undergoing pancreatic resections, SVV was set at 25% in patients with cardiopulmonary comorbidities, and 20% in patients without.

Hypovolaemia was defined as a preload responsive state with either a perfusion pressure or SV below the patients individualised baseline value. Further, hypovolaemia was classified into “*absolute*” hypovolaemia, which was attributed to surgical blood loss, or “*relative*” hypovolaemia, where surgical bleeding was not evident. “*Absolut*e” hypovolaemia was estimated via the volume of blood in suction devices and by the weighing of surgical swabs and was treated with a 1–2 ml/kg fluid bolus. “*Relative*” hypovolaemia was considered when the SVV exceeded the above-mentioned SVV targets, but in the absence of surgical bleeding. In this scenario, treatment was with a vasopressor, with the physiological rationale that use of vasoconstrictors will also cause venoconstriction, which in turn will decrease venous capacitance, resulting in an increase in venous return to the right atrium, i.e., increasing preload to the right heart.

The type of fluid (crystalloid or colloid) was based on the discretion of the treating anaesthetist. No fluids were administered for “third space” or “insensible” losses. The assumption was that these losses would be replaced by the administration of intraoperative medications, including anaesthetic, analgesic, vasoactive, and antibiotic infusions. At completion of surgery, the EV1000 or HemoSphere® clinical platforms were disconnected from the FloTrac® catheter.

### 2.5 Key outcomes

The primary outcome was the effect of using a patient-specific, surgery-specific AHDM algorithm for achieving a TO, with adjustment using propensity score matching. The TO criteria were defined according to the “International Expert Delphi Consensus on Defining Textbook Outcome in Liver Surgery” (13) and the “Nationwide Analysis of a Novel Quality Measure in Pancreatic Surgery” (14) (Table 1).

**Table 1:** Primary outcome definition of textbook outcome.

Textbook outcome (liver surgery)	Textbook outcome (pancreatic surgery)
• Absence of a postoperative bile leak of grade B or C (according to the severity grading of the International Study Group of Liver Surgery) (23). • Absence of postoperative liver failure of grade B or C (according to the severity grading of the International Study Group of Liver Surgery) (24). • No major postoperative complications (Clavien–Dindo ≥III) (25). • No 90-day readmission due to surgery-related major complications (Clavien–Dindo ≥III). • No in-hospital or 90-day mortality. • Median hospital length of stay ≤7 days.	• Absence of a postoperative pancreatic fistula of grade B or C (according to the severity grading of the International Study Group of Pancreatic Surgery) (26). • Absence of a postoperative bile leak of grade B or C (according to the severity grading of the International Study Group of Liver Surgery) (23). • Absence of a postpancreatectomy haemorrhage of grade B or C (according to the severity grading of the International Study Group of Pancreatic Surgery) (27). • No major postoperative complications (Clavien–Dindo ≥III) (25). • No 90-day readmission due to surgery-related major complications (Clavien–Dindo ≥III). • No in-hospital or 90-day mortality. • Median hospital length of stay ≤14 days.

Secondary outcomes included the effect of the AHDM algorithm on each component of the TO definition, with adjustment using propensity score matching. These outcomes include: the absence of surgery-specific complications, absence of severe postoperative complications, hospital length of stay, avoidance of hospital readmission, and avoidance of mortality while in hospital or at 90 days postoperatively.

### 2.6 Definitions

Complications were defined and classified according to the European Perioperative Clinical Outcome definitions (28). Severe complications were defined as the development of a grade III or higher complication according to the Clavien–Dindo classification (25). Bile leak and liver failure were defined according to the severity grading of the International Study Group of Liver Surgery (23, 24). Postpancreatectomy haemorrhage and postoperative pancreatic fistula were defined according to the severity grading of the International Study Group of Pancreatic Surgery (26, 27). LOS was defined as the time from surgical wound closure until the patient was formally discharged from the acute hospital ward. Prolonged LOS for liver surgery was defined as a stay longer than that of the 50th percentile (29). Prolonged LOS for pancreatic surgery was defined as a stay longer than 14 days, in accordance with the definition provided by the “Nationwide Analysis of a Novel Quality Measure in Pancreatic Surgery” (14).

### 2.7 Statistical analysis

Statistical analysis was performed using IBM SPSS Statistics software for Windows, version 23 (IBM Corporation, NY, USA) and R version 4.2.3 (R Development Core Team, Vienna, Austria, 2023). Before undertaking statistical analysis, the normality of the continuous variables was confirmed via a visual check of normal Q–Q plots and histograms. If normality was violated, non-parametric statistical methods were applied for the variable. Extreme values were also checked with the first and third quartiles and the step of the twofold interquartile range. All extreme values were reconciled with the original values of the data source.

Missing values analysis was undertaken before performing statistical analysis. After verifying the data with the data source, variables with a missing rate over 5% were identified. We then evaluated the missing patterns and whether the missing values occurred at random. In cases where the mechanism of missing data was not missing completely at random or missing at random, sensitivity analysis was also planned.

Statistical analysis was performed by grouping patients according to whether AHDM was used during intraoperative anaesthesia management. Student's *t*-test, Mann–Whitney *U* test, chi-square test, and Cochran–Armitage trend test were applied. Data were presented with mean ± standard deviation, median (first–third quartiles) or number of cases (percentile) for the descriptive statistics, and any estimated values are described with a 95% confidence interval (CI). Statistical results are presented with *p*-values and corresponding effect sizes.

Propensity score matching and weighting methods were planned to control the effects of covariates and reduce possible biases. Covariates included age, body mass index, age-adjusted Charlson's comorbidity index, hospital, surgery type, operation time, intraoperative blood loss, preoperative haemoglobin, platelet count, creatinine, and albumin concentrations. These were identified by correlation analysis or by their clinical relevance (Supplementary Table S2).

Cases with missing values for the propensity score match variables were excluded, and complete cases were used for regression analysis. The expected probability of logistic regression with AHDM was used as a dependent variable and other possible confounding parameters were used as independent variables to determine the propensity score of each case. The balance of propensity scores across the groups (AHDM group and Usual care group) was evaluated via a visual check of propensity score distribution. Optimal and nearest neighbourhood matching methods with 1:1, 2:1, and various ratios were evaluated using callipers of 0.1 or 0.2, if applicable. In addition, the inverse probability treatment weighting (IPTW) was also evaluated to control the confounding parameters by applying the weight (Supplementary Table S3).

The standardised difference was estimated to evaluate the balance of covariates after matching or weighting the data by propensity score. We considered that the matched or weighted data were well balanced when the standardised differences were smaller than 0.1. Regarding the values and covariate balance figures of the standardised differences, the weighted data with IPTW showed the balanced covariates between the AHDM group and the Usual care group. To reduce the impact of extreme weights on the parameters, we stabilised and truncated the weights at the 1st and 99th percentiles. At the end of the analysis, the planned sensitivity analysis was performed with various situations of complete case analysis (Supplementary Table S4).

The average effect of AHDM use in achieving a TO was evaluated using a weighted chi-square test. The effect of AHDM use on other results, such as postoperative complications, surgery-specific complications, LOS, ICU admission, and ICU LOS, was evaluated using a weighted chi-square test, weighted Cochran–Armitage trend test, weighted *t*-test, and weighted Mann–Whitney *U* test. A two-tailed *p*-value of <0.05 was considered statistically significant based on the null hypothesis significance testing, and the magnitude of effects was evaluated using the estimated effect sizes.

## 3 Results

**Figure 2:**
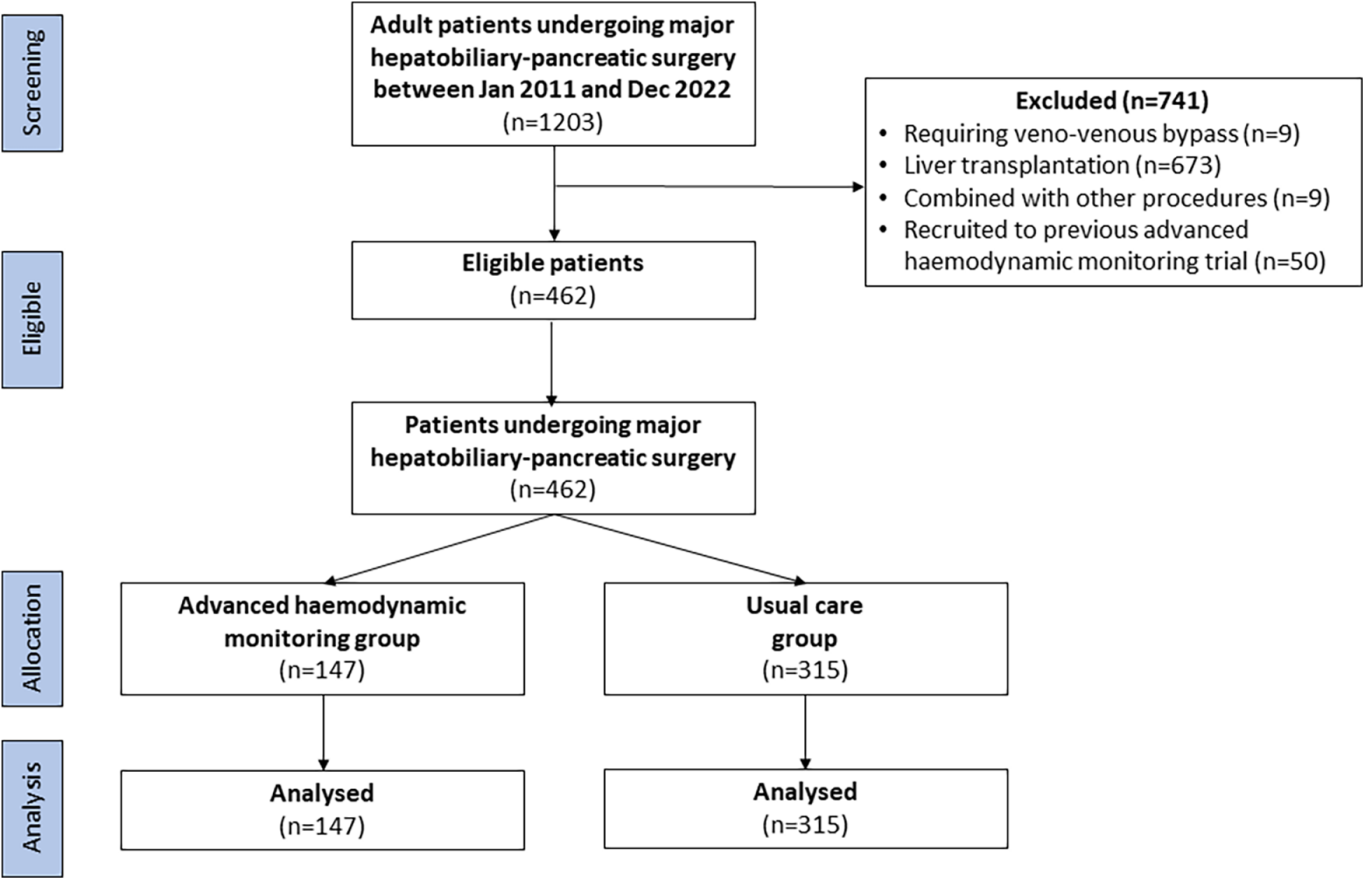
Study flow diagram.

In total, 1,203 patients underwent liver and pancreatic resection surgery at the three hospitals during the study period. Patient exclusions are summarised in the study flow diagram (see Figure 2). There were 462 patient cases that were included in the propensity score evaluation. Regarding the balance of the possible confounding parameters, weighted analysis was performed using IPTW (see Supplementary Table S5). With the IPTW method and truncated weights, the effective sample size (weighted sample size) was 780 cases. The demographic data, perioperative variables, and possible confounding parameters are presented in Table 2.

**Table 2:** Demographic data and possible confounding parameters.

			Unweighted						IPTW					
			Usual care group (*N* = 315)	AHDM group (*N* = 147)					Usual care group (*N* = 398)	AHDM group (*N* = 382)				
		Missing rate (%)	Mean ± SD	Mean ± SD	Mean difference	*p*-value	Effect size	Standardised difference	Mean ± SD	Mean ± SD	Mean difference	*p*-value	Effect size	Standardised difference
Preoperative	Age (years)	0.0	61.4 ± 12.7	62.2 ± 12.7	−0.8 (−3.3 to 1.7)	0.531	0.06	0.06	61.7 ± 12.6	62.2 ± 12.3	−0.5 (−2.2 to 1.3)	0.608	0.04	0.03
	Female gender	0.0	110 (34.9%)	67 (45.6%)	—	0.028^a^	0.10	0.22^b^	146 (36.7%)	158 (41.4%)	—	0.181	0.05	0.10^b^
	Body mass index (kg/m^2^)	0.0	27.19 ± 6.2	26.5 ± 5.32	0.69 (−0.47 to 1.86)	0.243	0.12	0.12^b^	26.82 ± 6.08	26.69 ± 5.46	0.13 (−0.68 to 0.94)	0.751	0.02	0.00
	Age-adjusted Charlson Comorbidity Index	0.0	4.1 ± 1.5	4.1 ± 1.5	0 (−0.3 to 0.3)	0.835	0.02	0.02	4.2 ± 1.5	4.2 ± 1.6	−0.1 (−0.3 to 0.1)	0.464	0.05	0.05
	Hospital	0.0				<0.001^a^	0.32					0.988	0.01	
	Hospital 1		13 (4.1%)	16 (10.9%)				(reference)	24 (6)	24 (6.3)				(reference)
	Hospital 2		11 (3.5%)	31 (21.1%)				0.56^b^	38 (9.5)	36 (9.4)				0.00
	Hospital 3		291 (92.4%)	100 (68%)				0.64^b^	336 (84.4)	322 (84.3)				0.00
	Operation group	0.2				0.291	0.09					0.980	0.02	
	Major liver		85 (27%)	44 (30.1%)				(reference)	102 (25.6%)	98 (25.6%)				(reference)
	Minor liver		105 (33.3%)	41 (28.1%)				0.11^b^	111 (27.9%)	102 (26.6%)				−0.02
	Whipple		114 (36.2%)	51 (34.9%)				0.03	163 (41%)	161 (42%)				0.01
	Other pancreatic		11 (3.5%)	10 (6.8%)				0.15^b^	22 (5.5%)	22 (5.7%)				0.00
	Haemoglobin (g/L)	0.0	133.9 ± 17.9	131.5 ± 17.7	2.5 (−1 to 6)	0.165	0.14	0.14^b^	133.4 ± 19.1	133.5 ± 18.6	−0.1 (−2.7 to 2.6)	0.948	0.00	0.01
	Platelet (×10^9^/L)	8.2	263.4 ± 113.2	248.8 ± 113.6	14.6 (−8.9 to 38.1)	0.222	0.13	0.13^b^	259.8 ± 111.6	255.6 ± 109.2	4.2 (−11.3 to 19.8)	0.593	0.04	−0.04
	Creatinine (μmol/L)	5.6	75.1 ± 30.5	77.4 ± 28.5	−2.2 (−8.3 to 3.9)	0.473	0.07	0.08	76.8 ± 36.8	77.8 ± 28.2	−0.9 (−5.6 to 3.7)	0.691	0.03	0.03
	Albumin (g/L)	8.4	36.7 ± 5.5	36.7 ± 5	0 (−1.1 to 1.1)	0.995	0.00	0.00	36.7 ± 5.2	36.8 ± 5	−0.1 (−0.8 to 0.6)	0.797	0.02	0.02
Intraoperative	Blood loss (ml)	0.0	300 (150–400) [0:3,500]	300 (150–400) [20:3,000]	—	0.07	0.10	0.06	300 (250–400) [0:3,500]	300 (150–400) [20:3,000]	—	0.004^a^	0.14	0.00
	Operation time (min)	0.6	411 ± 168.5	410 ± 186.6	0.9 (−33.4 to 35.2)	0.957	0.01	0.01	421.8 ± 168.6	413.8 ± 169	8 (−15.7 to 31.7)	0.509	0.05	−0.03

Data are expressed as mean ± SD, median (1st–3rd Quartile) [Min:Max], or number (%). Statistical analysis was performed using Student's *t*-test and Mann–Whitney *U* test for continuous variables, and Chi-square test for categorical variables. Effect size: Cohen's *d* for Student's *t*-test, *r* for Mann–Whitney *U* test, and Cramer's *V* for Chi-square test.

^a^ Two-sided *p*-value < 0.050.

^b^ The absolute standardised difference ≥0.1.

### 3.1 Primary outcome

The incidence of textbook outcomes is summarised in Table 3. Of the 780 weighted cases, 477 (61.2%, 95% CI: 57.7%–64.6%) achieved the TO. Patients in the AHDM group had a higher rate of TO [*n* = 259 (67.8%)] than those in the Usual care group [*n* = 218 (54.8%)], which was statistically significant (*χ*^2^ = 13.925, *p* < 0.001, Crémer's *V* = 0.13), and the estimated odds ratio (OR) was 1.74 (1.30–2.33).

**Table 3:** Incidence of textbook outcomes. Data presented as number (proportion).

	Unweighted				IPTW			
	Usual care group (*N* = 315)	AHDM group (*N* = 147)	*p*-value	OR (95% CI)	Usual care group (*N* = 398)	AHDM group (*N* = 382)	*p*-value	OR (95% CI)
Textbook outcome achieved	163 (51.7%)	94 (63.9%)	0.014^a^	1.65 (1.11–2.47)	218 (54.8%)	259 (67.8%)	<0.001^a^	1.74 (1.3–2.33)
Absence of surgery-specific complications	285 (90.5%)	140 (95.2%)	0.079	2.11 (0.9–4.91)	358 (90.2%)	365 (95.5%)	0.004^a^	2.34 (1.3–4.21)
Absence of severe complications (Clavien–Dindo ≥ 3)	271 (86.0%)	136 (92.5%)	0.045^a^	2.01 (1–4.01)	343 (86.4%)	352 (91.9%)	0.014^a^	1.79 (1.12–2.85)
Absence of readmission	294 (93.3%)	142 (96.6%)	0.156	2.03 (0.75–5.49)	371 (93.2%)	367 (96.1%)	0.077	1.78 (0.93–3.4)
Absence in-hospital or postoperative 90 days death	313 (99.4%)	146 (99.3%)	>0.99	0.93 (0.08–10.37)	395 (99.2%)	382 (100%)	0.249	—
Absence of prolonged length of hospital stay	174 (55.2%)	100 (68%)	0.009^a^	1.72 (1.14–2.6)	233 (58.5%)	276 (72.1%)	<0.001^a^	1.83 (1.35–2.46)

Data are presented as number (%). Chi-squared or Fisher's exact test was performed. Surgery-specific complications include: absence of postoperative bile leak of grade B or C, absence of postoperative liver failure of grade B or C for hepatic surgery, absence of postoperative pancreatic fistula (Grade B or C), absence of bile leak (grade B or C), absence of postpancreatectomy haemorrhage (grade B/C) for Whipple and Pancreatic surgeries. CVD indicates Clavien–Dindo postoperative complication grade. Length of hospital stay less than 8 hospital days for hepatic surgery, and 15 hospital days for Whipple and Pancreatic surgeries considered as a textbook outcome. Chi-square test was used.

^a^ Two-sided *p*-value <0.050.

### 3.2 Secondary outcome

**Figure 3:**
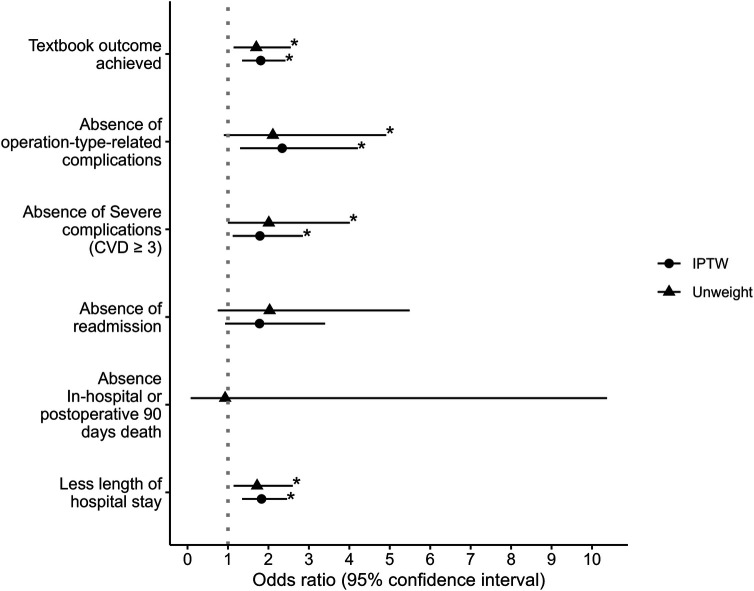
Odds ratios and 95% confidence intervals of the effect of advanced haemodynamic monitoring use on textbook outcome and components of textbook outcome. Note that the odds ratio of IPTW in absence in-hospital or postoperative 90 days death is blank due to the condition of being incomputable. * represents two-sided *p*-value ≤0.05.

Regarding the individual components of the TO, the AHDM group had a lower rate of surgery-specific complications, severe complications, and a shorter hospital LOS [ORs = 2.34 (95% CI: 1.30–4.21), 1.79 (95% CI: 1.12–2.85), and 1.83 (95% CI: 1.35–2.46), respectively]. There was no significant difference between the groups for hospital readmission and mortality (Table 3 and Figure 3). The postoperative complications are presented in Table 4. There was no difference observed between the groups for the development of any complication (*p* = 0.152) or the number of complications (*p* = 0.227). Detailed postoperative complications are presented in Supplementary Table S6. Postoperative ICU admission rate was significantly higher for the AHDM group [370 (96.6%)] than for the Usual care group [370 (93.2%), *p* = 0.031, OR = 2.08 (95% CI: 1.06–4.09)], but the estimated effect size was small (Crémer's *V* = 0.08; see Table 5). Hospital LOS was significantly longer for the Usual care group compared with the AHDM group, but the estimated effect size was small (*p* < 0.001, common language effect size *r* = 0.2; see Table 5).

**Table 4:** Postoperative complications.

		Unweighted					IPTW				
		Usual care group (*N* = 315)	AHDM group (*N* = 147)	*p*-value	Cremer's V	OR (95% CI)	Usual care group (*N* = 398)	AHDM group (*N* = 382)	*p*-value	Cremer's V	OR (95% CI)
Any complication		258 (81.9%)	108 (73.5%)	0.037^a^	0.1	0.61 (0.38–0.97)	327 (82.2%)	299 (78.1%)	0.150	0.05	0.77 (0.54–1.1)
Severe complication (Clavien–Dindo ≥ III)		44 (14%)	11 (7.5%)	0.045^a^	0.09	0.5 (0.25–1)	54 (13.6%)	31 (8.1%)	0.008^a^	0.09	0.53 (0.33–0.85)
No. of complications	Not complicated	57 (18.1%)	39 (26.5%)	0.020^a^	0.21	—	71 (17.8%)	84 (21.9%)	0.363	0.16	
	1 Complication	76 (24.1%)	30 (20.4%)				113 (28.4%)	81 (21.1)			
	2 Complications	56 (17.8%)	32 (21.8%)				66 (16.6%)	87 (22.7%)			
	3 Complications	46 (14.6%)	20 (13.6%)				52 (13.1%)	51 (13.3%)			
	4 Complications	2 (0.6%)	7 (4.8%)				5 (1.3%)	17 (4.4%)			
	>4 Complications	78 (24.8%)	19 (12.9%)				91 (22.9%)	63 (16.4%)			
CVD grade	Not complicated	57 (18.1%)	39 (26.5%)	0.045^a^	0.13	—	71 (17.8%)	84 (21.9%)	0.320	0.16	
	I	102 (32.4%)	41 (27.9%)				143 (35.9%)	104 (27.2%)			
	II	112 (35.6%)	56 (38.1%)				130 (32.7%)	164 (42.8%)			
	III	25 (7.9%)	6 (4.1%)				31 (7.8%)	17 (4.4%)			
	IV	17 (5.4%)	4 (2.7%)				20 (5%)	14 (3.7%)			
	V	2 (0.6%)	1 (0.7%)				3 (0.8%)	0 (0%)			

Data are presented as number (%). CVD indicates Clavien–Dindo postoperative complication grade. Chi-square test or Cochran–Armitage trend test was used.

^a^ Two-sided *p*-value <0.050.

**Table 5:** Intraoperative management and postoperative course.

	Unweighted					IPTW				
	Usual care group (*N* = 315)	AHDM group (*N* = 147)	Mean difference	*p*-value	Effect size	Usual care group (*N* = 398)	AHDM group (*N* = 382)	Mean difference	*p*-value	Effect size
Total fluid administration volume (ml)	2989 ± 1,773.7	2,357.8 ± 1,535.2	631.2 (297.2–965.2)	<0.001^a^	0.37	2,872.2 ± 1,771.2	2,628.4 ± 1,677.4	243.8 (1.2–486.4)	0.049^a^	0.14
Vasopressor use	115 (36.5%)	48 (32.7%)	—	0.419	0.04	157 (39.4%)	140 (36.6%)	—	0.405	0.03
Norepinephrine use	72 (22.9%)	36 (24.5%)	—	0.699	0.02	99 (24.9%)	92 (24.1%)	—	0.797	0.01
Norepinephrine dose (µg)^b^	735 (408.75–1,574.25), [20:9,600]	1,230 (720–2,610) [18:7,320]	—	0.052	0.19	720 (440–1,585.36) [20:9,600]	960 (600–2,640) [18:7,320]	—	0.103	0.12
Ephedrine use	60 (19%)	16 (10.9%)		0.027	0.1	80 (20.1%)	60 (15.7%)		0.106	0.06
Ephedrine dose (mg)^b^	12 (7.5–18), [3:50]	26.25 (7.875–30) [3:60]	—	0.047^a^	0.23	12 (6.63–18) [3:50]	30 (8.57–30) [3:60]	—	<0.001^a^	0.32
ICU admission	290 (92.1%)	143 (97.3%)	—	0.031^a^	0.1	370 (93.2%)	370 (96.6%)	—	0.031^a^	0.08
ICU stay duration (days)	1 (1–1.5), [0:21]	1 (1–3) [0:30]	—	<0.001^a^	0.21	1 (1–2) [0:20]	1 (1–2) [0:30]	—	0.444	0.04
Length of hospital stay (days)	9 (6–15), [2:142]	8 (6–11) [3:45]	—	0.013^a^	0.14	9 (6–15) [2:122]	8 (6–11) [3:45]	—	<0.001^a^	0.2

Data are presented as mean ± SD, number (%), or median (1st–3rd quartile) [Min:Max]. Student’s *t*-test, Chi-square test, or Mann–Whitney *U* test was used. Effect size is Cohen's *d*, OR, or common language effect size *r* for Mann–Whitney *U* test.

^a^ Two-sided *p*-value <0.050.

^b^ In cases of using an indexed vasopressor.

## 4 Discussion

### 4.1 Key findings

In this multicentre, retrospective, propensity score–matched study, we investigated the effects of an AHDM algorithm on achieving TO for patients undergoing HBP surgery. We studied the implementation of a patient-specific, surgery-specific algorithm that sought to individualise fluid and vasoactive therapies to maintain circulatory homeostasis and end-organ perfusion. This approach respects the unique ventriculoarterial coupling relationship present in each individual patient, while tailoring therapy to the dynamic requirements of the circulation throughout each stage of surgery. We observed that the use of this algorithm was associated with an increase in the rate of TO, a reduced rate of severe complications, and a reduction in hospital LOS. Our findings support the inclusion of “perfusion pressure” and dynamic SVV thresholds as essential elements in AHDM for HBP surgery and support the use of moderate intraoperative fluid therapy for these patients. The use of this algorithm was safe, and our results support further prospective trials to evaluate the clinical effect of this treatment paradigm on long-term patient outcomes.

### 4.2 Relations to the literature

This is the first study to explore the impact of an AHDM algorithm on TO. A recent systematic review highlighted a wide range of TO rates in HBP surgery, from 15.8% to 69.1% (12). Our study aligns with the upper limit of this range, showing a TO rate of 54.8% for the Usual care group and 67.8% for the AHDM group. This finding demonstrates the potential for such treatment paradigms to improve patient-centred outcomes, and further studies are warranted.

Our institution has previously undertaken prospective, randomised, clinical trials in patients undergoing HBP surgeries using a protocolised AHDM algorithm (18, 21), similar to the algorithm used in the present study. However, both of these previous studies were inadequately powered to measure meaningful differences in postoperative complications, and neither evaluated the impact of the surgery-specific algorithm on textbook outcomes. In only one of these studies was the use of the AHDM algorithm associated with a reduction in the number of complications and LOS (21). Peltoniemi et al. (16) demonstrated fewer severe surgical complications, clinically relevant pancreatic fistulas, and shorter hospital LOS for patients undergoing AHDM for pancreatic surgery. While their algorithm differed from ours, where a crystalloid/albumin fluid bolus (3:1 ratio) was administered when SVV exceeded 12% or central venous oxygen saturation fell below 70%, both studies demonstrate a consistent decrease in LOS and severe complications in patients receiving AHDM.

A meta-analysis employing AHDM for non-cardiac surgery patients indicated that AHDM could lower postoperative complication rates, most commonly infections and anastomotic leakage (30). However, the effectiveness of AHDM in reducing mortality or shortening LOS remains unclear. While goal-directed therapy algorithms have been extensively studied in patients undergoing major surgery, multiple large, randomised, control trials have largely failed to demonstrate any significant difference in clinically meaningful patient-centred outcomes (30). While many of these algorithms aim to maximise a physiological endpoint or achieve a target derived from a cohort of patients, such targets may be entirely inappropriate for any given individual. Our AHDM algorithm respects that each patient requires a unique approach and supports the use of precision and personalised haemodynamic management for such patients.

Perioperative guidelines and goal-directed therapy algorithms frequently prescribe MAP or SBP targets in isolation to other haemodynamic parameters. However, in patients undergoing major surgery, there can be significant reductions in MAP occurring simultaneously to significant increases in CVP. The combined effect can lead to a critical reduction in the arteriovenous pressure gradient across vital organs below their auto-regulatory threshold, and end-organ hypoperfusion. The AHDM algorithm described in our study respects this physiological tenet by targeting a “perfusion pressure” across the systemic circulation. Previous research has described perfusion pressure as highly heterogeneous between patients and within the same patient over time, with lower perfusion pressures being more strongly associated with negative outcomes than MAP or CVP pressures individually (31, 32).

The goal-directed therapy algorithms employed in the OPTIMISE I and II trials attempted to maximise SV through repeated fluid boluses and inotropic support titration (33, 34). By contrast, the AHDM algorithm described in our study sought to individualise each patient's SV to their preoperative baseline values. Compared with our study, patients in the OPTIMISE I trial had markedly higher rates of complications despite greater fluid therapy administration in both intervention (7.1 vs. 5.3 ml/kg/h) and control arms (8.9 vs. 5.2 ml/kg/h), higher vasoactive therapy use and comparatively shorter operations (33). However, the OPTIMISE I trial was not specific to HBP surgery and numerous other methodological differences make direct comparison difficult.

Our AHDM algorithm permitted fluid boluses in response to the needs of the circulation, in accordance with an individualised SVV target that was dynamically adjusted for each patient. Prior research has demonstrated the benefits of accurate SVV measurements and a high target (SVV > 25%) in managing volume status during hepatic resection (35–38) and improving outcomes following pancreaticoduodenectomy (17, 21). While SVV monitoring has been correlated with decreased intraoperative bleeding (39), this association was not observed in the present study.

Our study had a relatively low incidence of complications related to excessive or insufficient fluid therapy, including pulmonary oedema, acute kidney injury, and surgical site infection. A large retrospective study of non-cardiac surgical patients found the optimal fluid therapy rate associated with the lowest incidence of complications was between 6 and 7 ml/kg/h (40). Our study varies only marginally from this reported optimal range. Compared with the RELIEF trial, which randomised patients to restrictive and liberal fluid therapy in major abdominal surgery, patients in both arms of our study received less fluid than either the restrictive (6.5 ml/kg/h) and liberal (10.9 ml/kg/h) groups, less vasoactive support than either the restrictive (81.7%) and liberal (78.2%) groups, and had longer durations of surgery (41). Despite this, the incidence of acute kidney injury and surgical site infection were both higher in the RELIEF trial than our study. It is important to note that the RELIEF trial excluded liver resection patients; however, their inclusion criteria ensured their overall patient population was at an increased risk of postoperative complications—a selection variable that was not considered in the present study.

Noradrenaline was the primary vasopressor administered in our study; its use is rationalised by its vasoconstrictive, ionotropic, and chronotropic effects, collectively enhancing systemic vascular resistance, venous return, and preload. By comparison, the RELIEF trial favoured the use of metaraminol as the primary vasopressor of choice. While early noradrenaline use has been associated with reduced fluid resuscitation and related complications (42), the optimal vasoactive agent for surgical patients remains an area of active research (43).

### 4.3 Strengths and limitations

Our study has several strengths. To date, it is the largest multicentre retrospective analysis reporting the effects of a patient-specific, surgery-specific AHDM algorithm on TO, enhancing the generalisability of our findings. The use of propensity score matching and IPTW helped mitigate potential confounding factors, enhancing the robustness of our results. Since this study was conducted in three hospitals, its external validity is applicable to other hospitals undertaking similar procedures. Finally, our findings provide foundational data for sample size calculations for future randomised, controlled trials in this area.

Our study also has several limitations. While we controlled for multiple variables, unmeasured confounders could still influence our results because of the retrospective nature of the study. The use of AHDM at the sample hospitals was at the discretion of the attending anaesthesiologist, introducing the possibility of selection bias. Despite our efforts to address missing data, the presence of missing values may introduce bias and affect the generalisability of our findings. Finally, given the retrospective nature of this study, we cannot establish a causal relationship between our AHDM algorithm and TO.

## 5 Conclusion

The intraoperative use of a patient-specific, surgery-specific AHDM algorithm during HBP surgery increased the rate of TO after surgery. Individualised AHDM was associated with reduced surgery-specific complications, severe postoperative complications, and hospital LOS. A larger, prospective, randomised control trial testing this AHDM algorithm's effect(s) on the outcome(s) in this patient cohort is justified.

## Data Availability

The raw data supporting the conclusions of this article will be made available by the authors, without undue reservation.
