# Supplementary material for: The effect of an intraoperative patient-specific, surgery-specific haemodynamic algorithm in improving textbook outcomes for hepatobiliary–pancreatic surgery: a multicentre retrospective study
Source: Front Surg. 2024 May 27;11:1353143. doi: 10.3389/fsurg.2024.1353143 (PMC11163073; doi:10.3389/fsurg.2024.1353143)
Supplement: Supplementary file 1 [file Datasheet1.docx]

Supplementary Material

**1 Supplementary figure and tables**

Supplementary File Table 1: Textbook Outcome Definitions for Hepatobiliary-Pancreatic Surgery

| **Authors** | **Study period** | **Indication** | **Components of textbook outcome** |
| --- | --- | --- | --- |
| Merath et al. (1) | 2013–2015 | Benign and malignant hepatopancreatic lesions | No complications. No prolonged length of stay. No 90-day mortality. No 90-day readmission. |
| Merath et al. (2) | 1993–2015 | Intrahepatic cholangiocarcinoma | R0 resection. No transfusion. No complications. No prolonged length of stay. No 30-day mortality. No 30-day readmission. |
| Tsilimigras et al. (3) | 2000–2015 | Hepatocellular carcinoma | R0 resection. No reoperation. No complications (Clavien–Dindo ≥III). No prolonged length of stay. No 90-day mortality. No 90-day readmission. |
| Heidsma et al. (4) | 2000–2016 | Pancreatic neuroendocrine tumours | R0 resection. No complications (Clavien–Dindo ≥III). No prolonged length of stay. No 90-day mortality. No 90-day readmission. |
| Azap et al. (5) | 2013–2017 | Benign and malignant hepatopancreatic lesions | No postoperative surgical complications. No prolonged length of stay. No 90-day readmission. No 90-day mortality. |
| Mehta et al. (6) | 2013–2015 | Malignant hepatopancreatic lesions | No complications. No prolonged length of stay. No 90-day mortality. No 90-day readmission. |
| Mehta et al. (7) | 2015–2017 | Malignant hepatopancreatic lesions | No complications. No prolonged length of stay. No 90-day mortality. No 90-day readmission. |
| International Expert Delphi Consensus (8) | 2020–2021 | Major liver surgery | No postoperative bile leak of grade B/C, postoperative liver failure grade B/C, or major postoperative complications (Clavien–Dindo ≥III). No 90-day readmission. No in-hospital or 90-day mortality. |
| Nationwide Analysis of a Novel Quality Measure (9) | 2014–2017 | Pancreatic surgery | No postoperative pancreatic fistula, bile leak, or postpancreatectomy haemorrhage (all grade B/C). No severe complications (Clavien–Dindo ≥III). No 30-day readmission. No in-hospital or 30-day mortality. No prolonged length of stay (defined as >14 days). |

1. Merath K, Chen Q, Bagante F, Alexandrescu S, Marques HP, Aldrighetti L, et al. A Multi-institutional International Analysis of Textbook Outcomes Among Patients Undergoing Curative-Intent Resection of Intrahepatic Cholangiocarcinoma. JAMA Surg. 2019;154(6):e190571. doi: 10.1001/jamasurg.2019.0571

2. Merath K, Chen Q, Bagante F, Beal E, Akgul O, Dillhoff M, et al. Textbook Outcomes Among Medicare Patients Undergoing Hepatopancreatic Surgery. Ann Surg. 2020;271(6):1116-23. doi: 10.1097/sla.0000000000003105

3. Tsilimigras DI, Mehta R, Merath K, Bagante F, Paredes AZ, Farooq A, et al. Hospital variation in Textbook Outcomes following curative-intent resection of hepatocellular carcinoma: an international multi-institutional analysis. HPB (Oxford). 2020;22(9):1305-13. doi: 10.1016/j.hpb.2019.12.005

4. Heidsma CM, Hyer M, Tsilimigras DI, Rocha F, Abbott DE, Fields R, et al. Incidence and impact of Textbook Outcome among patients undergoing resection of pancreatic neuroendocrine tumors: Results of the US Neuroendocrine Tumor Study Group. J Surg Oncol. 2020;121(8):1201-8. doi: 10.1002/jso.25900

5. Azap RA, Paredes AZ, Diaz A, Hyer JM, Pawlik TM. The association of neighborhood social vulnerability with surgical textbook outcomes among patients undergoing hepatopancreatic surgery. Surgery. 2020;168(5):868-75. doi: 10.1016/j.surg.2020.06.032

6. Mehta R, Paredes AZ, Tsilimigras DI, Moro A, Sahara K, Farooq A, et al. Influence of hospital teaching status on the chance to achieve a textbook outcome after hepatopancreatic surgery for cancer among Medicare beneficiaries. Surgery. 2020;168(1):92-100. doi: 10.1016/j.surg.2020.02.024

7. Mehta R, Tsilimigras DI, Pawlik TM. Assessment of Magnet status and Textbook Outcomes among medicare beneficiaries undergoing hepato-pancreatic surgery for cancer. J Surg Oncol. 2021;124(3):334-42. doi: 10.1002/jso.26521

8. Görgec B, Cacciaguerra AB, Pawlik TM, Aldrighetti LA, Alseidi AA, Cillo U, et al. An International Expert Delphi Consensus on Defining Textbook Outcome in Liver Surgery (TOLS). Annals of surgery. 2022. doi: 10.1097/sla.0000000000005668

9. van Roessel S, Mackay TM, van Dieren S, van der Schelling GP, Nieuwenhuijs VB, Bosscha K, et al. Textbook Outcome: Nationwide Analysis of a Novel Quality Measure in Pancreatic Surgery. Ann Surg. 2020;271(1):155-62. doi: 10.1097/sla.0000000000003451

Supplementary File Table 2: Correlation Analysis Between Collected Variables and Textbook Outcome

|  | Variables | Correlation coefficients | P value | Missing rate (%) |
| --- | --- | --- | --- | --- |
| Preoperative | Age (year) | -0.150 | 0.003* | 0.00 |
|  | Female gender | 0.052 | 0.298 | 0.00 |
|  | Body Mass Index (kg/m^2^) | 0.013 | 0.792 | 0.00 |
|  | Age-adjusted Charlson Comorbidity Index | -0.183 | <0.001* | 0.00 |
|  | Hospital | -0.014 | 0.777 | 0.00 |
|  | Operation group | -0.055 | 0.280 | 0.00 |
|  | Haemoglobin (g/L) | 0.134 | 0.008* | 0.00 |
|  | Platelet concentration (x10^9^/L) | -0.079 | 0.116 | 0.00 |
|  | Creatinine (μmol/L) | -0.037 | 0.467 | 0.00 |
|  | Albumin (g/L)_ | 0.127 | 0.012* | 0.00 |
|  | White blood cell count (x10^9^/L) | -0.133 | 0.008* | 0.76 |
|  | Sodium (mmol/L) | 0.042 | 0.687 | 76.20 |
|  | Bicarbonate (mmol/L) | -0.014 | 0.897 | 76.20 |
|  | Urea (mmol/L) | -0.082 | 0.155 | 24.30 |
|  | Estimated glomerular filtration rate (mL/min/1.73m^2^) | 0.016 | 0.765 | 12.15 |
|  | Bilirubin (μmol/l) | -0.019 | 0.736 | 15.95 |
|  | Aspartate transferase (U/L) | -0.064 | 0.578 | 80.25 |
|  | Alanine transaminase (U/L) | -0.024 | 0.799 | 71.90 |
|  | Alkaline phosphatase (U/L) | -0.020 | 0.837 | 72.91 |
|  | Gamma-glutamyl transferase (U/L) | -0.118 | 0.219 | 72.15 |
|  | International normalised ratio | -0.010 | 0.877 | 32.91 |
|  | Prothrombin time (s) | -0.202† | 0.042* | 74.18 |
|  | Activated partial thromboplastin clotting time (s) | -0.174 | 0.086 | 75.19 |
| Intraoperative | Blood loss (ml) | -0.006 | 0.905 | 0.00 |
|  | Urine output (ml) | -0.159 | 0.009* | 30.89 |
|  |  |  |  |  |
|  |  |  |  |  |
|  | Operation time (min) | -0.231† | <0.001* | 0.00 |
|  | Flotrac use | 0.125 | 0.013* | 0.00 |
| The results of Spearman correlation analysis. *: indicates 2-sided P <0.05, †: indicates absolute correlation coefficient > 0.2. | | | | |

**Supplementary File Table 3:** The balance of propensity scores across the groups.

Histogram of propensity scores. The distributions of propensity scores of both groups overlap throughout the range. The calculated propensity scores commonly support both groups.


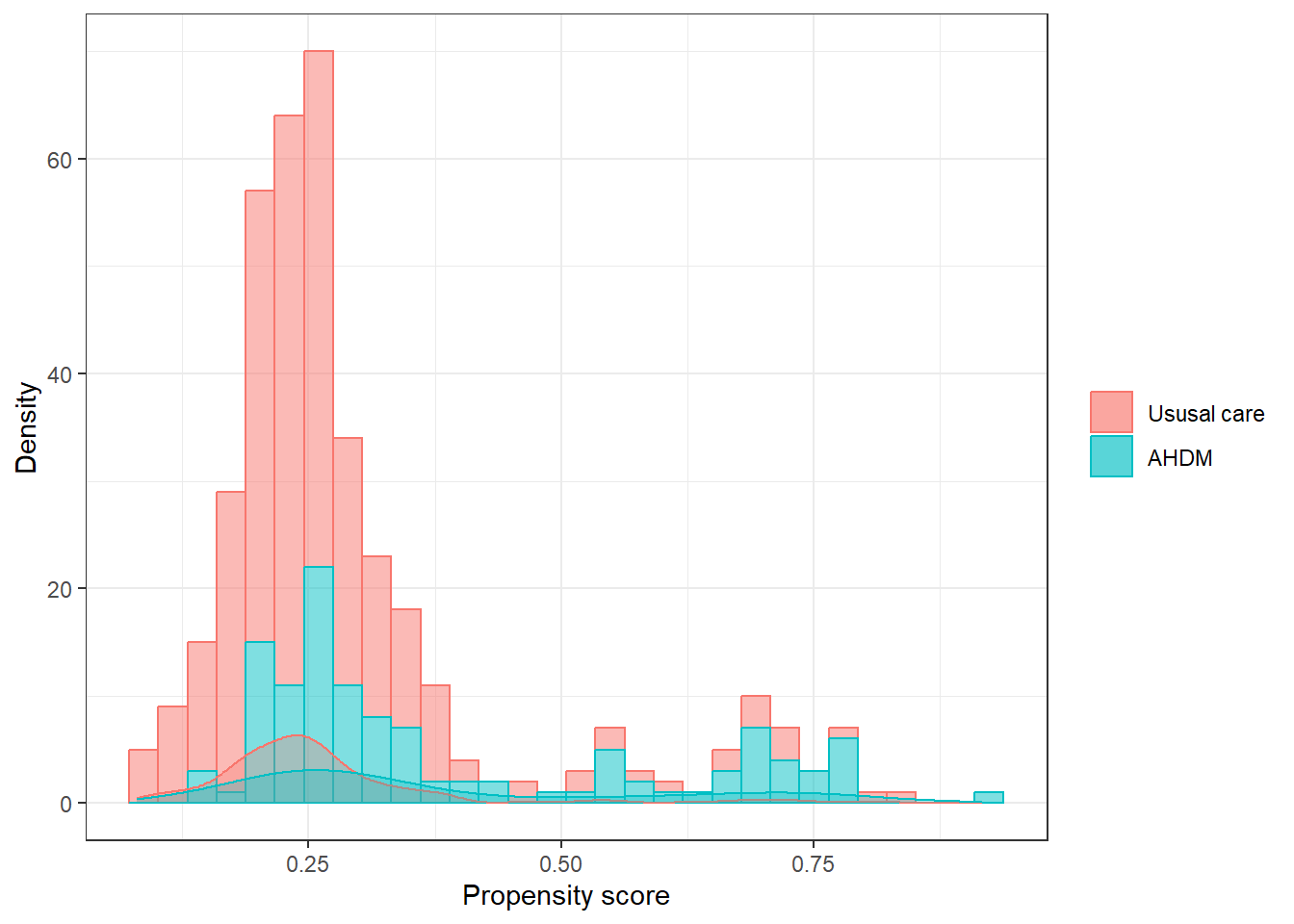


**Quantiles of the propensity score.**

| Probability of quantile | Usual care group | Advance haemodynamic monitoring group | difference |
| --- | --- | --- | --- |
| 0.00 | 0.08 | 0.134 | -0.055 |
| 0.10 | 0.164 | 0.203 | -0.039 |
| 0.20 | 0.188 | 0.232 | -0.044 |
| 0.25 | 0.208 | 0.249 | -0.041 |
| 0.30 | 0.227 | 0.267 | -0.04 |
| 0.40 | 0.24 | 0.284 | -0.044 |
| 0.50 | 0.255 | 0.334 | -0.078 |
| 0.60 | 0.273 | 0.427 | -0.154 |
| 0.70 | 0.303 | 0.642 | -0.339 |
| 0.75 | 0.37 | 0.723 | -0.353 |
| 0.80 | 0.825 | 0.917 | -0.092 |
| 0.90 | 0.08 | 0.134 | -0.055 |
| 1.00 | 0.164 | 0.203 | -0.039 |

Note that the propensity score differences at high probability range are considerably increasing compared to those at low probability. During IPTW, we stabilized and truncated the weights at the 1^st^ and 99^th^ percentiles to reduce the impact of extreme weights on the parameters.

**Supplementary File Table 4:** Sensitivity analysis result

|  | **IPTW without preoperative albumin**  **(N = 831)** | | | | **IPTW without preoperative creatinine**  **(N = 784)** | | | | **IPTW without preoperative platelet**  **(N = 803)** | | | |
| --- | --- | --- | --- | --- | --- | --- | --- | --- | --- | --- | --- | --- |
| **The textbook outcome achieved (in total)** | **512 (61.2 (95%CI: 57.9 ‒ 64.5))** | | | | **497 (61.6 (95%CI: 58.2 ‒ 64.9))** | | | | **487 (61.6 (95%CI: 58.3 ‒ 65))** | | | |
|  | **Usual care**  **group** | **AHDM**  **group** | **P value** | **OR** | **Usual care**  **group** | **AHDM**  **group** | **P value** | **OR** | **Usual care**  **group** | **AHDM**  **group** | **P value** | **OR** |
| **No. of cases** | 425 | 406 | - | - | 399 | 385 | - | - | 409 | 394 | - | - |
| **Textbook outcome achieved** | 231 (54.5) | 273 (67.2) | <0.001* | 1.71 (1.29 - 2.27) | 216 (54.1) | 261 (67.8) | <0.001* | 1.78 (1.33 - 2.38) | 223 (54.5) | 267 (67.6) | <0.001* | 1.74 (1.31 - 2.32) |
| **Absence of operation-type-related complications** | 384 (90.4) | 384 (94.6) | 0.021* | 1.86 (1.09 - 3.19) | 360 (90.2) | 368 (95.6) | 0.004* | 2.35 (1.3 - 4.22) | 370 (90.5) | 377 (95.7) | 0.004 | 2.34 (1.3 - 4.21) |
| **Absence of severe complications (Clavien-Dindo ≥ 3)** | 367 (86.6) | 371 (91.4) | 0.027* | 1.65 (1.06 - 2.57) | 345 (86.5) | 355 (92.2) | 0.009* | 1.85 (1.16 - 2.96) | 354 (86.8) | 365 (92.4) | 0.009 | 1.86 (1.16 - 2.97) |
| **Absence of readmission** | 396 (93.4) | 386 (95.1) | 0.301 | 1.36 (0.76 - 2.46) | 372 (93.2) | 371 (96.1) | 0.073 | 1.8 (0.94 - 3.43) | 382 (93.4) | 379 (96.2) | 0.075 | 1.79 (0.94 - 3.41) |
| **Absence In-hospital or postoperative 90 days death** | 422 (99.3) | 401 (98.8) | 0.497 | 0.57 (0.14 - 2.4) | 396 (99.2) | 385 (100) | 0.249 | - | 406 (99.3) | 394 (100) | 0.249 | - |
| **Less length of hospital stay** | 247 (58.1) | 294 (72.4) | <0.001* | 1.89 (1.41 - 2.53) | 231 (57.9) | 277 (71.9) | <0.001* | 1.87 (1.38 - 2.51) | 238 (58.2) | 283 (71.6) | <0.001* | 1.82 (1.35 - 2.44) |

# Supplementary File Table 5: Evaluation of the covariates balance after matching or weighting by the propensity score. Absolute standardized differences in corresponding matching or weighting methods.

|  | **Matching or weighting methods** | | | | | | | | | | |
| --- | --- | --- | --- | --- | --- | --- | --- | --- | --- | --- | --- |
| **Parameters** | **Unmatched** | **Optimal 1:1** | **Optimal 2:1** | **NNM 1:1** | **NNM 2:1** | **NNM 1:1 caliper 0.1** | **NNM 2:1 caliper 0.1** | **NMM 1:1 caliper 0.2** | **NNM 2:1 caliper 0.2** | **NNM variable ratio** | **IPTW** |
| Age | 0.06 | -0.07 | 0.06 | 0.04 | 0.04 | -0.04 | 0.01 | -0.03 | 0.03 | -0.1 | 0.03 |
| Age-adjusted Charlton Comorbidity Index | 0.02 | -0.09 | 0.02 | 0.1 | 0.1* | -0.05 | -0.03 | 0.01 | 0 | -0.11* | 0.05 |
| Operation time | 0.01 | 0.15* | 0.11* | 0.1* | 0.05 | 0.01 | -0.02 | 0.06 | 0.02 | 0.13* | -0.03 |
| Intraoperative blood loss | 0.06 | -0.07 | -0.05 | 0 | -0.05 | -0.1* | -0.01 | -0.09 | -0.02 | -0.03 | 0 |
| Body Mass Index | 0.14* | -0.04 | -0.17* | -0.25* | -0.28* | 0.03 | 0.05 | 0 | 0.03 | -0.08 | 0.01 |
| Preoperative haemoglobin | 0.13* | -0.04 | -0.06 | -0.06 | -0.02 | 0.05 | 0.11* | 0 | 0.03 | 0 | -0.04 |
| Preoperative platelet | 0.08 | -0.04 | 0.1* | 0.14* | 0.12* | -0.02 | -0.03 | 0.07 | 0.05 | -0.03 | 0.03 |
| Preoperative creatinine | 0 | -0.01 | 0 | -0.06 | -0.04 | -0.05 | -0.02 | -0.08 | -0.04 | 0.05 | 0.02 |
| Preoperative albumin | - | - | - | - | - | - | - | - | - | - | - |
| Hospital 1 (reference) | 0.56* | 0.13* | 0.18* | 0.14* | 0.18* | 0.02 | 0.02 | 0.03 | 0.03 | 0.13* | 0 |
| Hospital 2 | 0.64* | -0.14* | -0.23* | -0.17* | -0.24* | -0.01 | -0.01 | -0.01 | -0.01 | -0.14* | 0 |
| Hospital 3 | - | - | - | - | - | - | - | - | - | - | - |
| Surgery type: Major liver (reference) | 0.11* | 0.01 | -0.08 | -0.03 | -0.07 | 0.01 | -0.01 | 0.01 | 0 | -0.01 | -0.02 |
| Surgery type: Minor liver | 0.03 | -0.13* | -0.03 | 0 | -0.01 | -0.04 | -0.03 | -0.04 | -0.02 | -0.08 | 0.01 |
| Surgery type: Whipple | 0.15* | 0.03 | 0.04 | 0.01 | 0.04 | 0.01 | 0.01 | -0.01 | -0.02 | 0.02 | 0 |
| Surgery type: Other pancreatic | 0.06 | -0.07 | 0.06 | 0.04 | 0.04 | -0.04 | 0.01 | -0.03 | 0.03 | -0.1 | 0.03 |
| No. of usual care group | 315 | 276 | 119 | 238 | 119 | 238 | 98 | 179 | 100 | 183 | 398 |
| No. of AHDM group | 147 | 119 | 119 | 119 | 119 | 119 | 98 | 98 | 100 | 100 | 382 |
| Total No. | 462 | 395 | 238 | 357 | 238 | 357 | 196 | 277 | 200 | 283 | 780 |

**Supplementary File figure 1:** Covariate balance graphs of various matching and weighting methods. The dotted line indicates a 0.1 threshold of standardized difference. The covariates are balanced with the IPTW method.

| Optimal, 1:1 ratio | Optimal, 2:1 ratio |
| --- | --- |
| 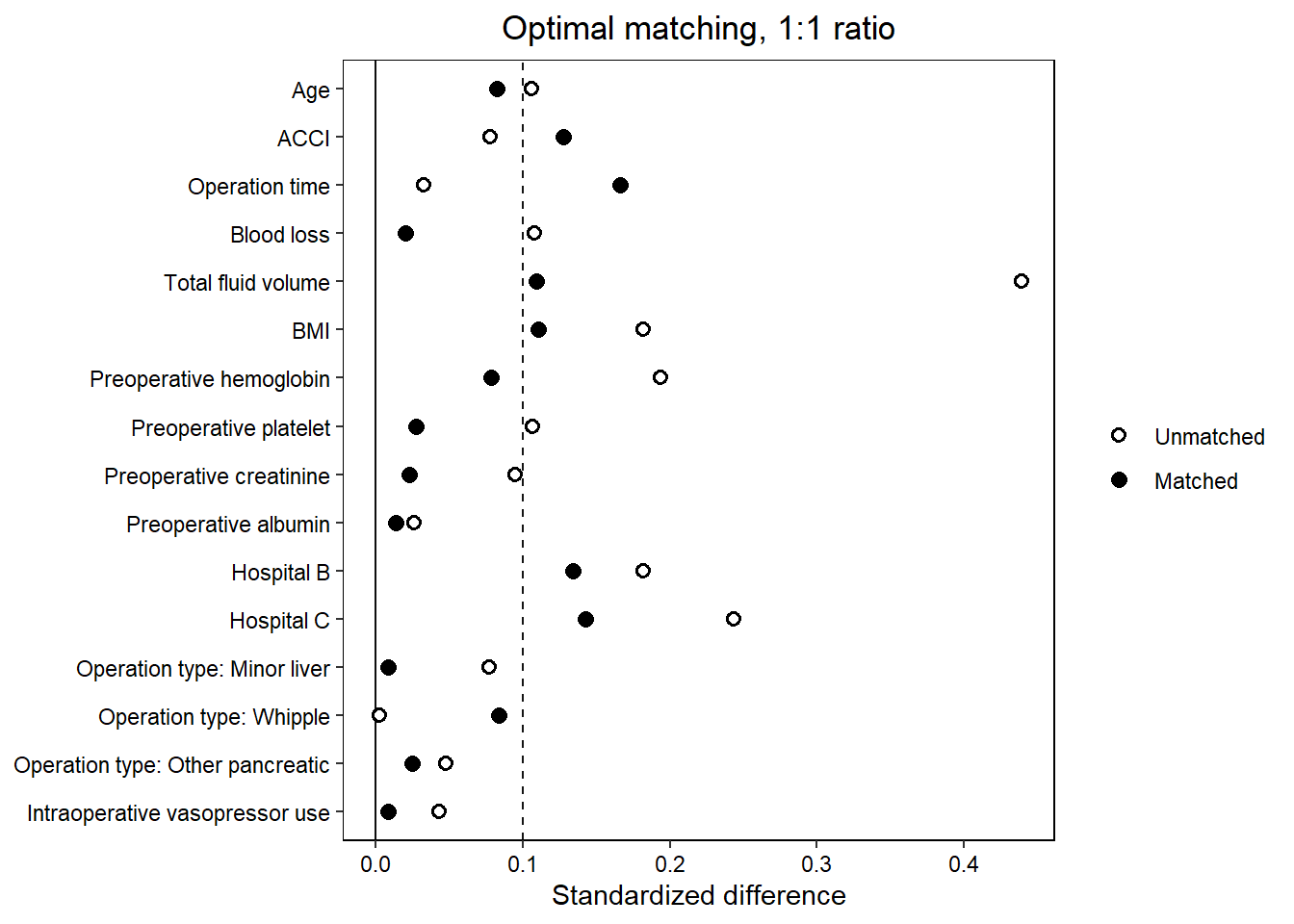 | 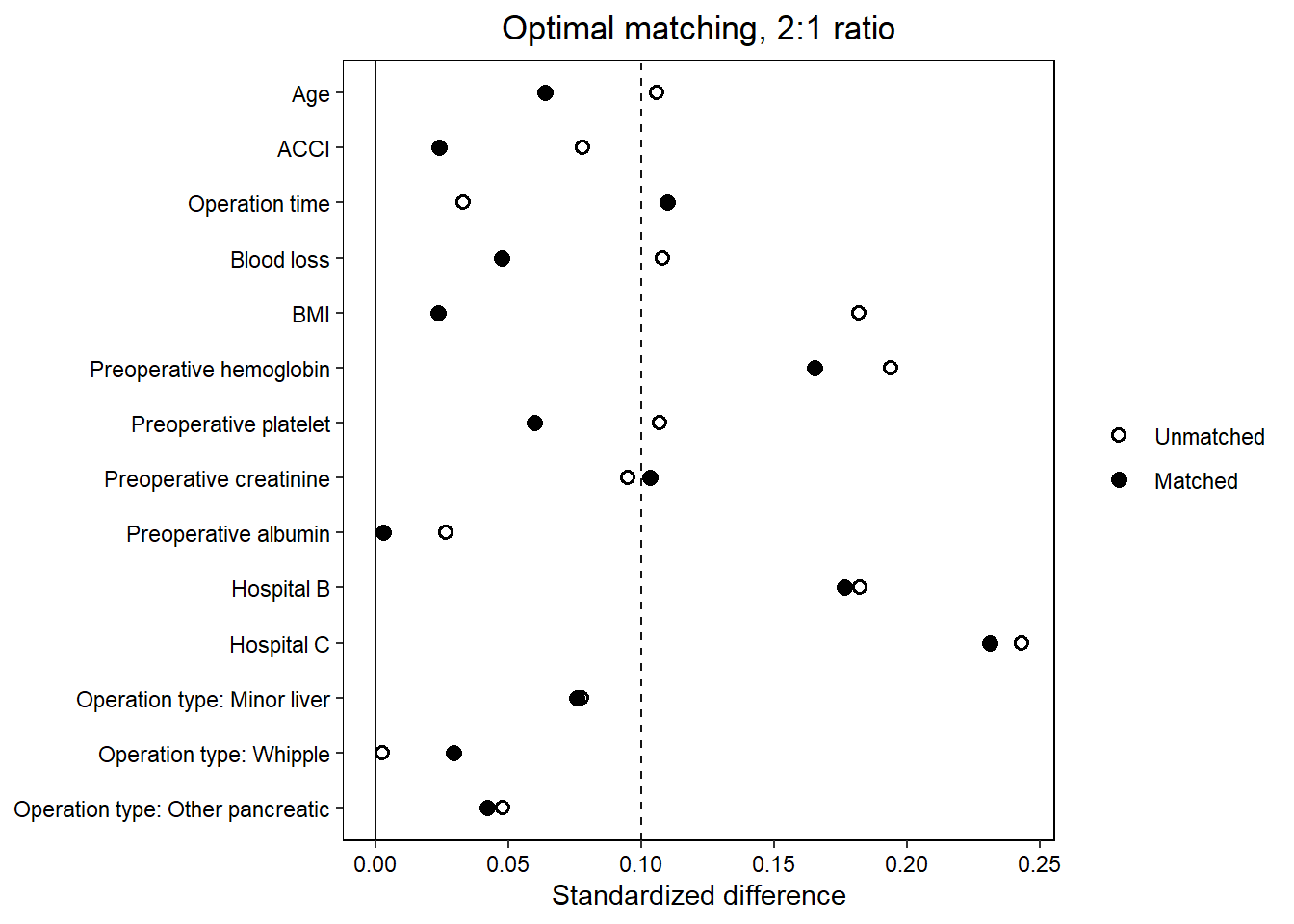 |
| NNM, 1:1 ratio, Mahalanobis | NNM, 2:1 ratio, Mahalanobis |
| 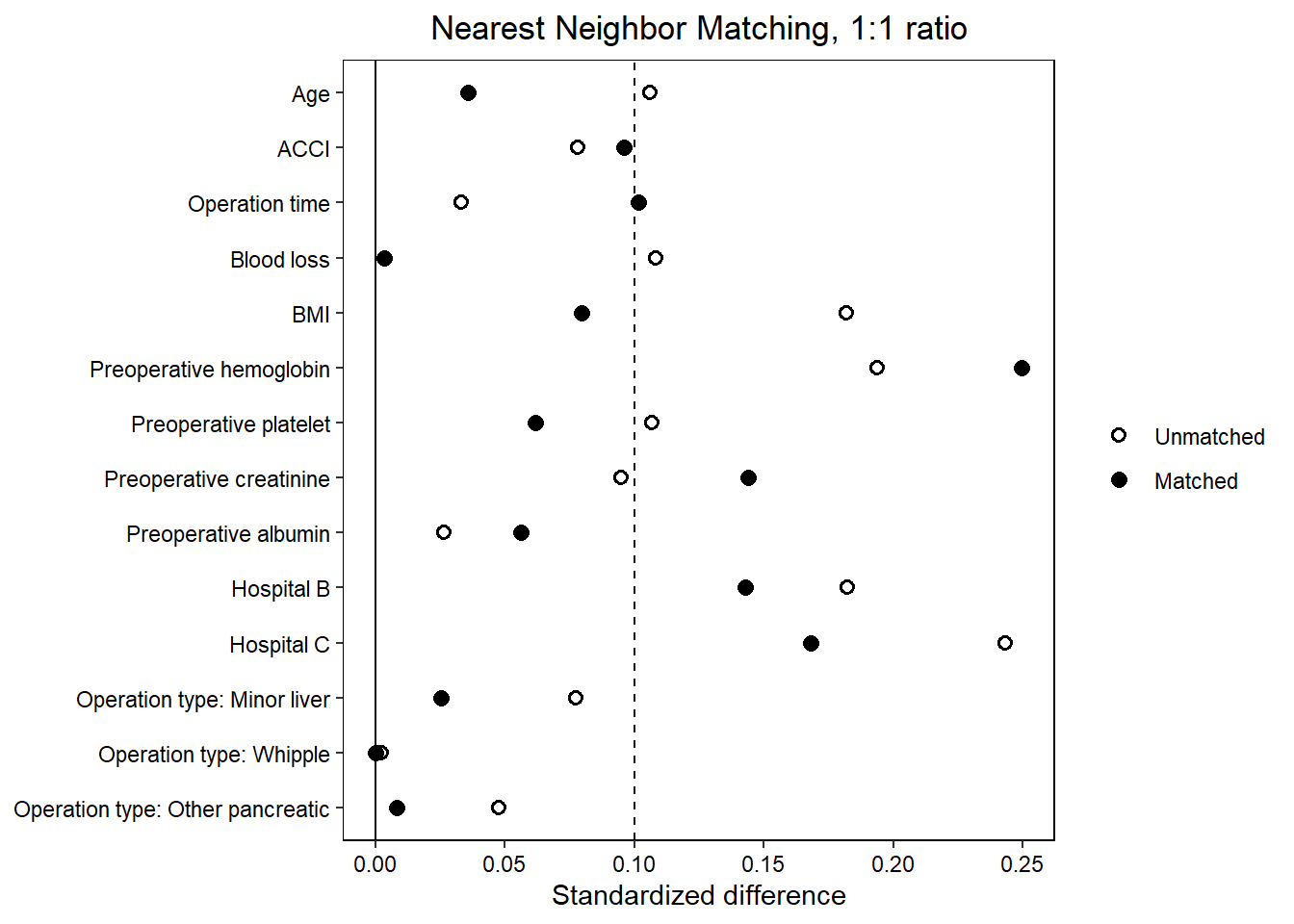 | 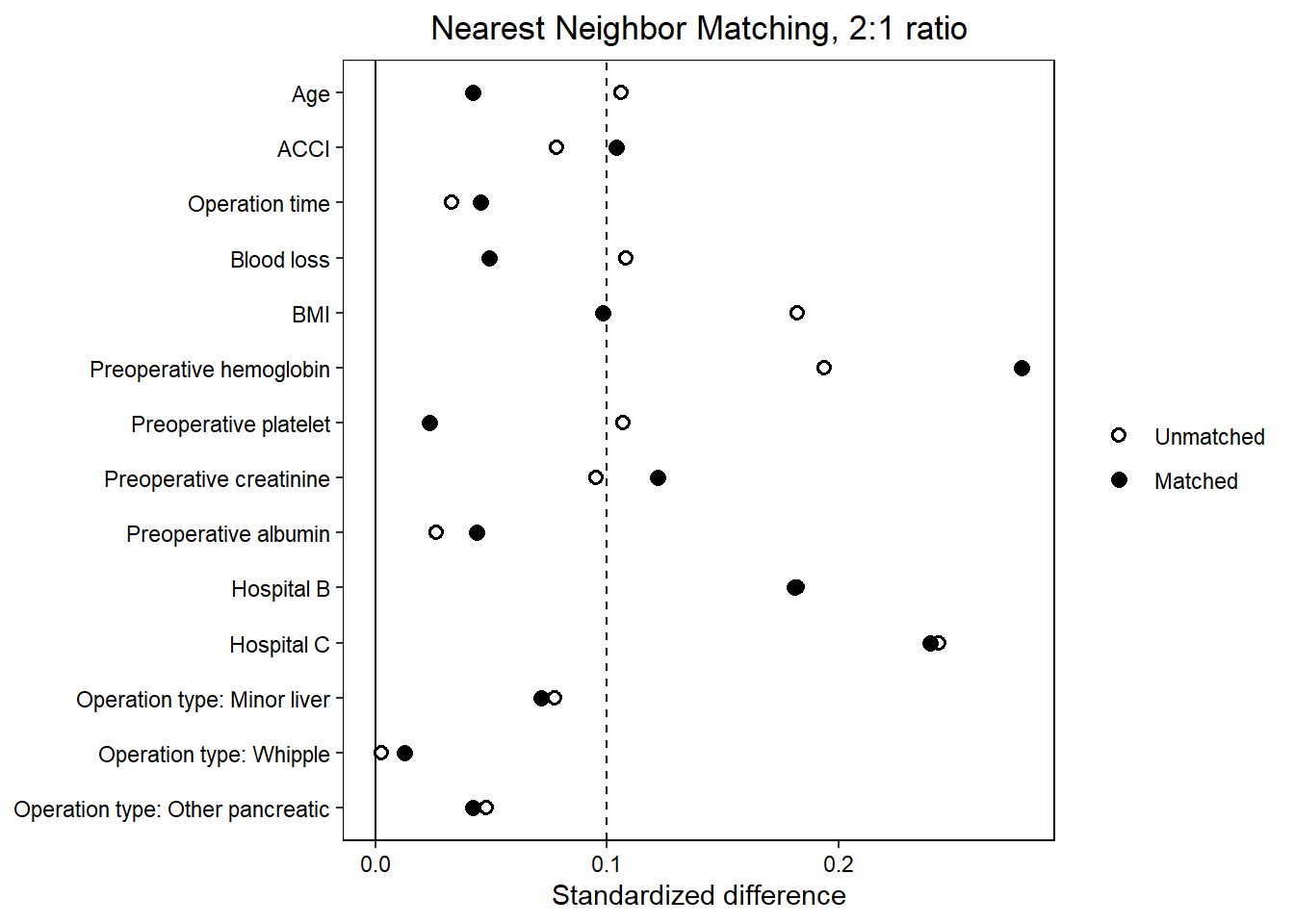 |
| NNM, 1:1 ratio, caliper 0.1 | NNM, 2:1 ratio, caliper 0.1 |
| 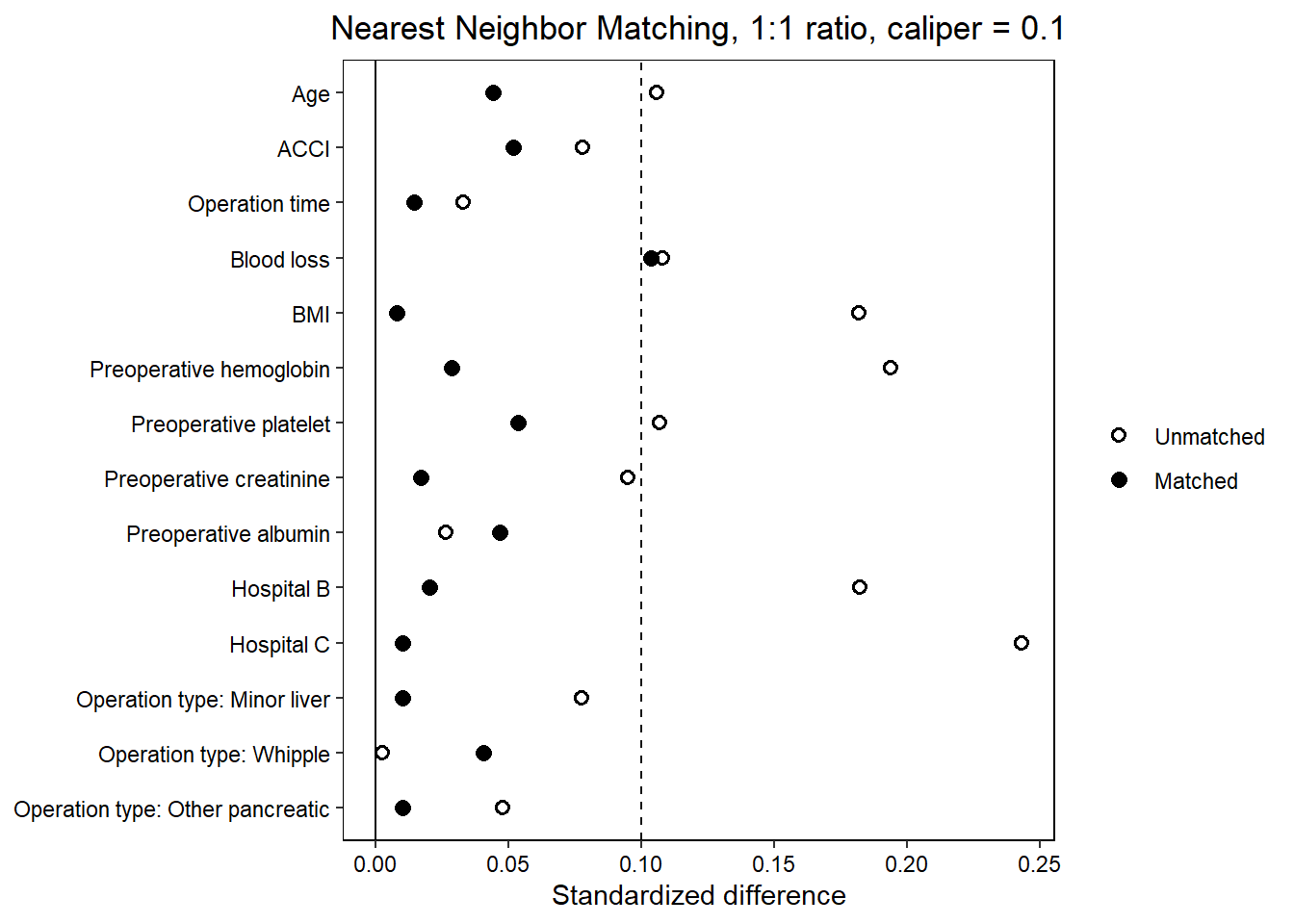 | 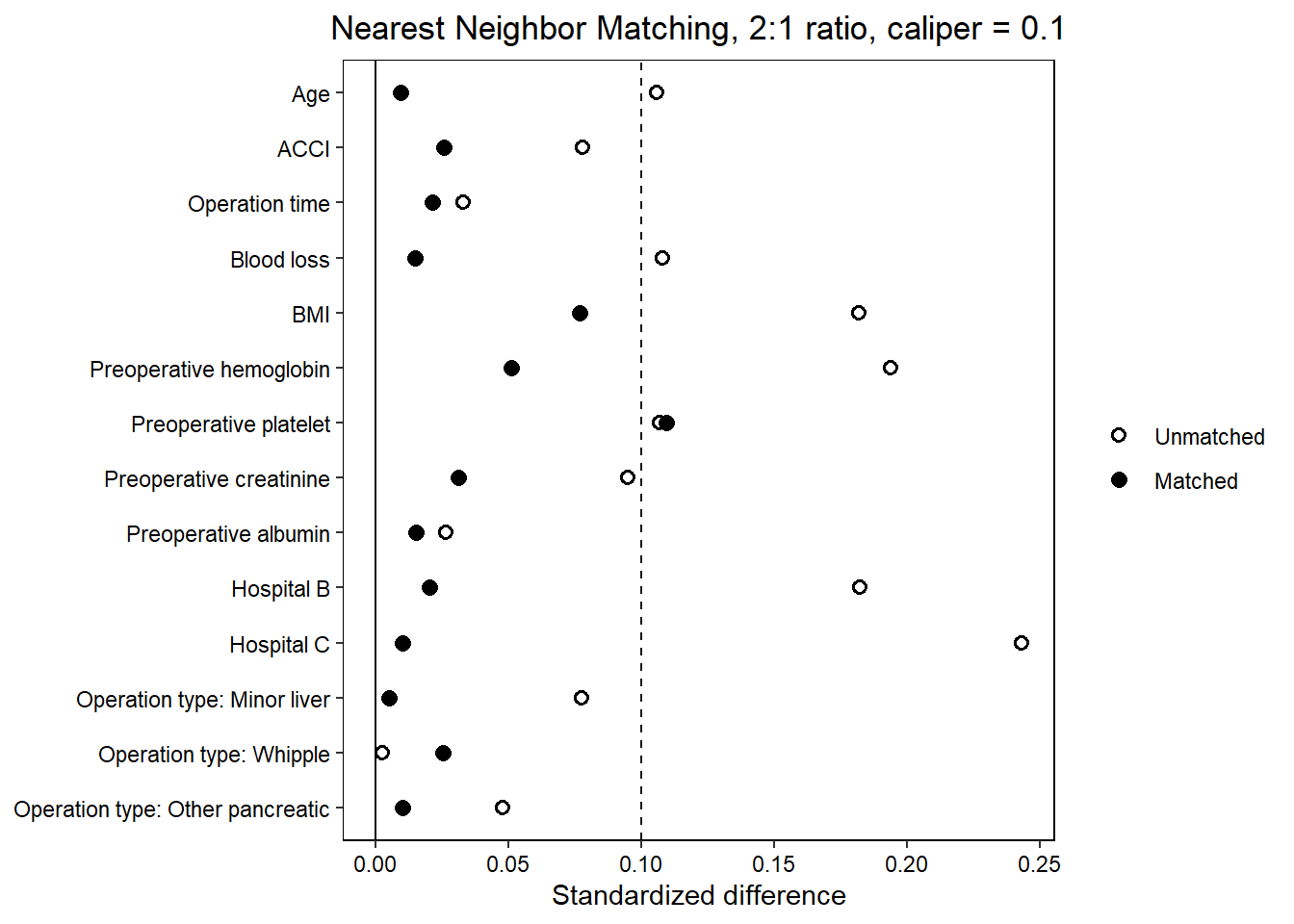 |
| NNM, 1:1 ratio, caliper 0.2 | NNM, 2:1 ratio, caliper 0.2 |
| 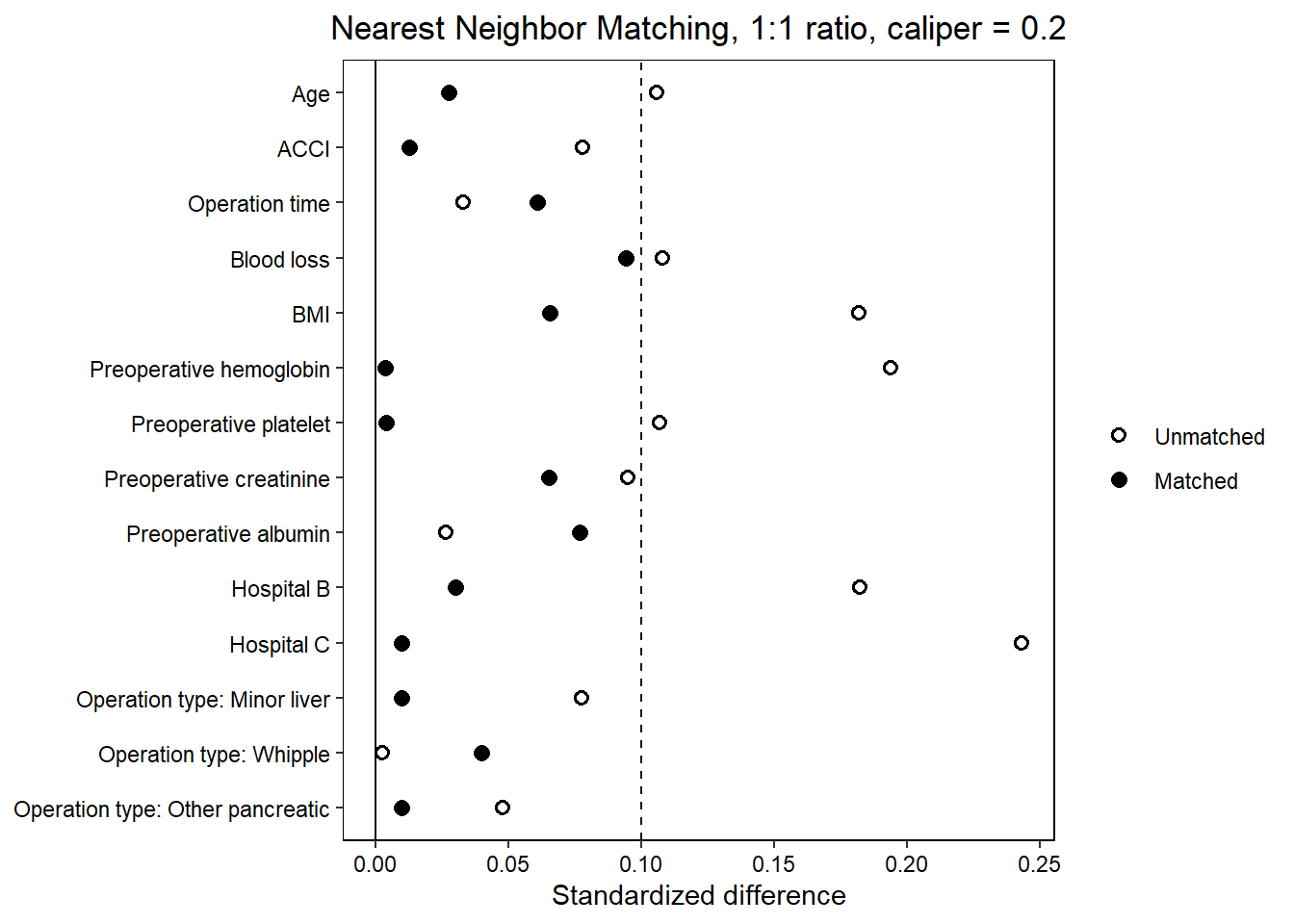 | 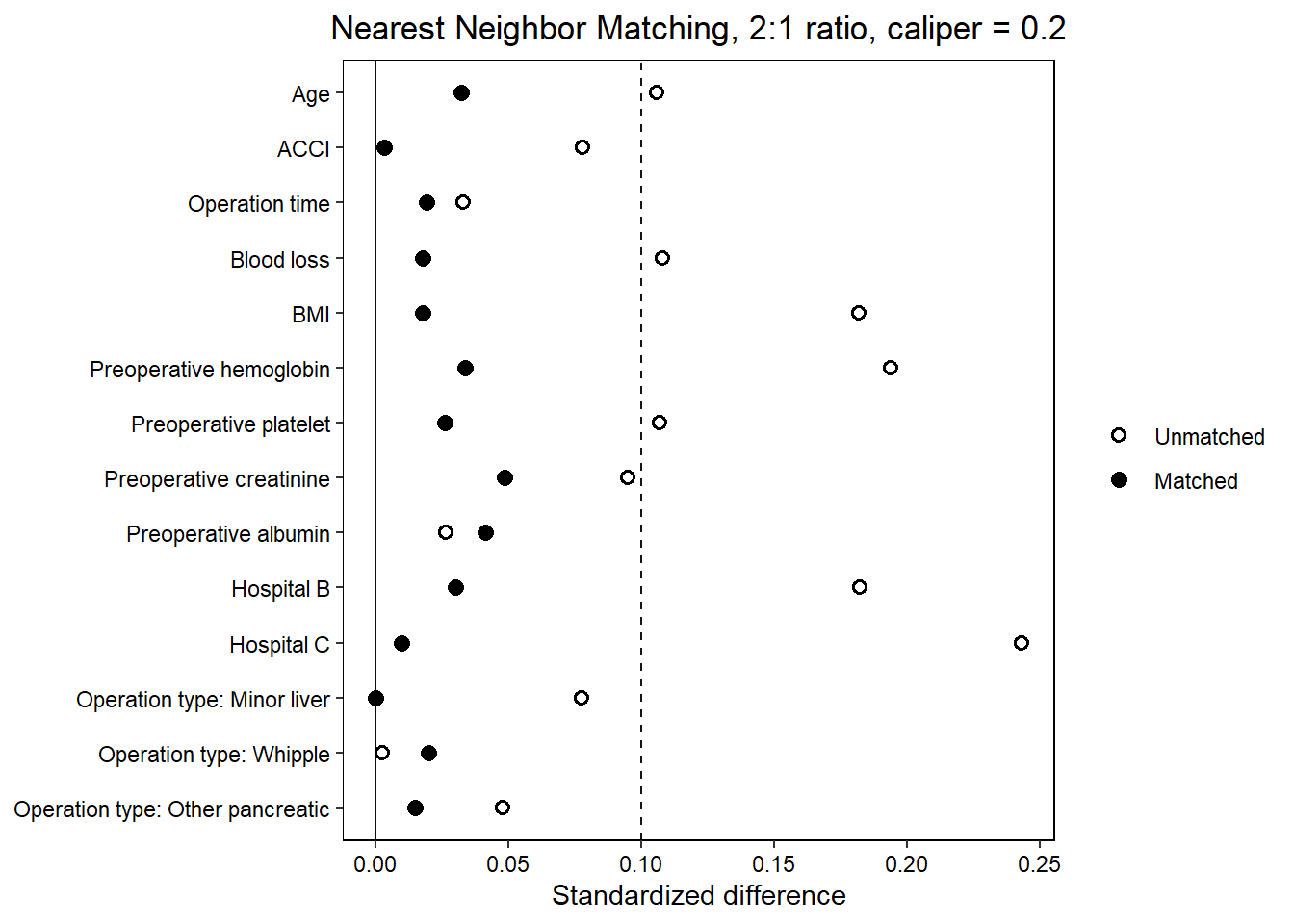 |
| NNM, variable ratio, Mahalanobis | IPTW |
| 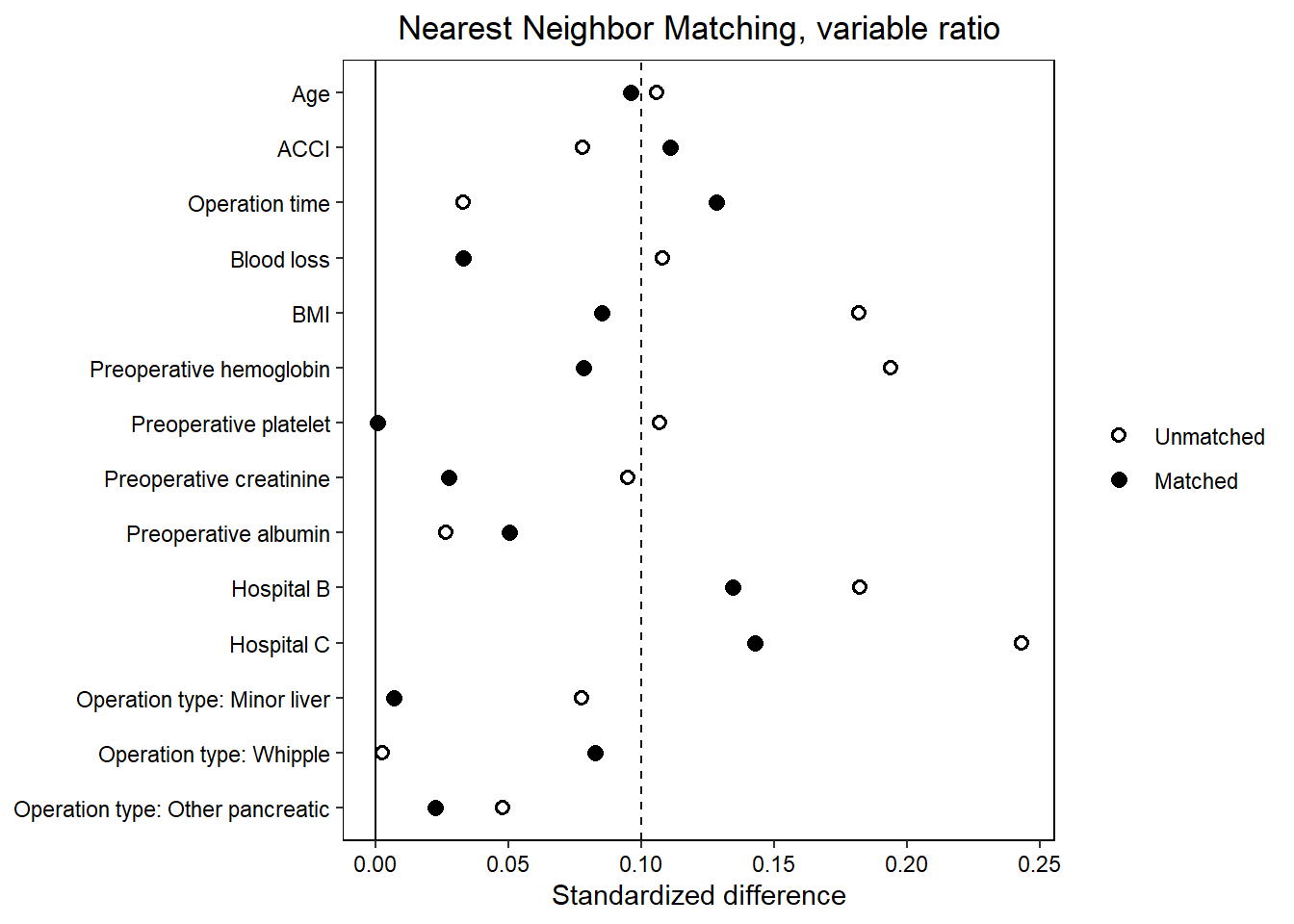 | 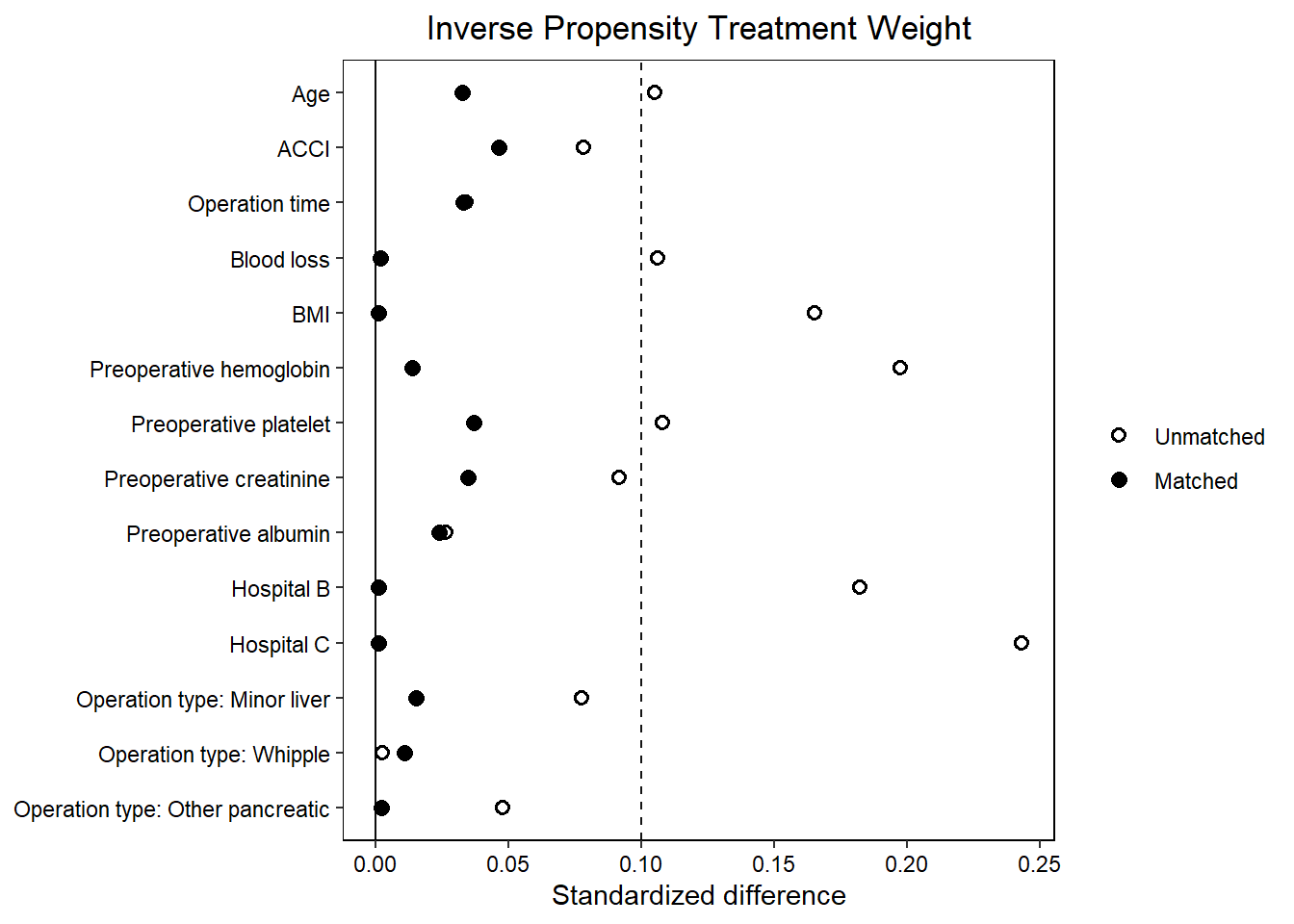 |

Supplementary File Table 6: Detailed Postoperative Complications

|  | **Specific complications** | **Unweighted** | | **IPTW** | |
| --- | --- | --- | --- | --- | --- |
|  |  | **Usual care**  **Group (N = 315)** | **AHDM**  **Group (N = 147)** | **Usual care**  **group (N = 398)** | **AHDM**  **group (N = 382)** |
| **Hepatic surgery** | Bile leakage (Grade B or C) | 5 (2.6) | 1 (1.2) | 5 (2.3) | 0 (0) |
|  | Delayed gastric emptying (Grade B or C) | 8 (4.2) | 0 (0) | 10 (4.7) | 0 (0) |
|  | Post-liver resection haemorrhage (Grade B or C) | 4 (2.1) | 2 (2.3) | 5 (2.3) | 7 (3.5) |
|  | Liver insufficiency (Grade B or C) | 4 (2.1) | 2 (2.3) | 4 (1.9) | 2 (1) |
|  | Other surgery-specific complications | 2 (1.1) | 1 (1.2) | 1 (0.5) | 4 (2) |
|  | Surgery related death | 0 (0) | 1 (1.2) | 0 (0) | 0 (0) |
|  | Readmission | 0 (0) | 0 (0) | 0 (0) | 0 (0) |
|  | Sepsis | 3 (1.6) | 2 (2.3) | 3 (1.4) | 0 (0) |
|  | Surgical site infection - deep organ space | 18 (9.5) | 0 (0) | 15 (7) | 0 (0) |
|  | Acute kidney injury (more than grade 2) | 5 (2.6) | 0 (0) | 7 (3.3) | 0 (0) |
|  | Myocardial infarction | 0 (0) | 1 (1.2) | 0 (0) | 3 (1.5) |
|  | Cerebrovascular accident | 0 (0) | 0 (0) | 0 (0) | 0 (0) |
|  | Other cardiovascular complications | 4 (2.1) | 1 (1.2) | 5 (2.3) | 0 (0) |
|  | Pulmonary oedema | 2 (1.1) | 0 (0) | 1 (0.5) | 0 (0) |
|  | Respiratory failure | 6 (3.2) | 3 (3.5) | 6 (2.8) | 4 (2) |
|  | Pulmonary embolism | 3 (1.6) | 1 (1.2) | 2 (0.9) | 1 (0.5) |
|  | Other haemorrhage | 0 (0) | 1 (1.2) | 0 (0) | 0 (0) |
|  | Pneumonia | 11 (5.8) | 0 (0) | 9 (4.2) | 0 (0) |
|  | Other medical complications | 7 (3.7) | 1 (1.2) | 9 (4.2) | 0 (0) |
| **Pancreatic surgery** | Postoperative pancreatic fistula (Grade B or C) | 12 (9.6) | 1 (1.6) | 16 (8.6) | 4 (2.2) |
|  | Post-pancreatectomy haemorrhage (Grade B or C) | 5 (4) | 2 (3.2) | 7 (3.8) | 7 (3.8) |
|  | Bile leakage (Grade B or C) | 10 (8) | 1 (1.6) | 14 (7.6) | 4 (2.2) |
|  | Delayed gastric emptying (Grade B or C) | 28 (22.4) | 5 (8.1) | 39 (21.1) | 16 (8.7) |
|  | Pancreatic insufficiency | 2 (1.6) | 1 (1.6) | 3 (1.6) | 4 (2.2) |
|  | Liver insufficiency (Grade B or C) | 2 (1.6) | 0 (0) | 3 (1.6) | 0 (0) |
|  | Other surgery-specific complications | 5 (4) | 1 (1.6) | 7 (3.8) | 4 (2.2) |
|  | Surgery related death | 2 (1.6) | 0 (0) | 3 (1.6) | 0 (0) |
|  | Readmission | 17 (13.6) | 3 (4.8) | 23 (12.4) | 13 (7.1) |
|  | Sepsis | 14 (11.2) | 1 (1.6) | 19 (10.3) | 2 (1.1) |
|  | Surgical site infection - deep organ space | 12 (9.6) | 5 (8.1) | 16 (8.6) | 11 (6) |
|  | Acute kidney insufficiency (more than grade 2) | 1 (0.8) | 1 (1.6) | 1 (0.5) | 4 (2.2) |
|  | Myocardial infarct | 0 (0) | 0 (0) | 0 (0) | 0 (0) |
|  | Other cardiovascular complications | 4 (3.2) | 0 (0) | 5 (2.7) | 0 (0) |
|  | Cerebrovascular accident | 2 (1.6) | 1 (1.6) | 3 (1.6) | 5 (2.7) |
|  | Pulmonary oedema | 0 (0) | 0 (0) | 0 (0) | 0 (0) |
|  | Respiratory failure | 5 (4) | 0 (0) | 7 (3.8) | 0 (0) |
|  | Pulmonary embolism | 1 (0.8) | 3 (4.8) | 1 (0.5) | 11 (6) |
|  | Other haemorrhage | 0 (0) | 0 (0) | 0 (0) | 0 (0) |
|  | Pneumonia | 9 (7.2) | 2 (3.2) | 13 (7) | 6 (3.3) |
|  | Other medical complications | 4 (3.2) | 1 (1.6) | 5 (2.7) | 2 (1.1) |
